# Network Pharmacology Combined with Machine Learning to Reveal the Action Mechanism of Licochalcone Intervention in Liver Cancer

**DOI:** 10.3390/ijms242115935

**Published:** 2023-11-03

**Authors:** Fangfang Guo, Xiaotang Yang, Chengxiang Hu, Wannan Li, Weiwei Han

**Affiliations:** Key Laboratory for Molecular Enzymology and Engineering of Ministry of Education, School of Life Science, Jilin University, 2699 Qianjin Street, Changchun 130012, China; guoff15@mails.jlu.edu.cn (F.G.); yangxt22@mails.jlu.edu.cn (X.Y.); hucx23@mails.jlu.edu.cn (C.H.)

**Keywords:** network pharmacology, machine learning, quantitative calculation, flavonoids, licorice

## Abstract

There are reports indicating that licochalcones can inhibit the proliferation, migration, and invasion of cancer cells by promoting the expression of autophagy-related proteins, inhibiting the expression of cell cycle proteins and angiogenic factors, and regulating autophagy and apoptosis. This study aims to reveal the potential mechanisms of licochalcone A (LCA), licochalcone B (LCB), licochalcone C (LCC), licochalcone D (LCD), licochalcone E (LCE), licochalcone F (LCF), and licochalcone G (LCG) inhibition in liver cancer through computer-aided screening strategies. By using machine learning clustering analysis to search for other structurally similar components in licorice, quantitative calculations were conducted to collect the structural commonalities of these components related to liver cancer and to identify key residues involved in the interactions between small molecules and key target proteins. Our research results show that the seven licochalcones molecules interfere with the cancer signaling pathway via the NF-κB signaling pathway, PDL1 expression and PD1 checkpoint pathway in cancer, and others. Glypallichalcone, Echinatin, and 3,4,3′,4′-Tetrahydroxy-2-methoxychalcone in licorice also have similar structures to the seven licochalcones, which may indicate their similar effects. We also identified the key residues (including ASN364, GLY365, TRP366, and TYR485) involved in the interactions between ten flavonoids and the key target protein (nitric oxide synthase 2). In summary, we provide valuable insights into the molecular mechanisms of the anticancer effects of licorice flavonoids, providing new ideas for the design of small molecules for liver cancer drugs.

## 1. Introduction

Cancer seriously affects human health and is expected to become the main cause of death in every country/region in the 21st century [1]. According to calculations for 2020, liver cancer is the sixth most common cancer and the third leading cause of cancer death [2]. Scientists from the International Agency for Research on Cancer (IARC) and its collaborating institutions have estimated the global burden of cancer in 2020 and predicted that by 2040, the number of new cases and deaths will increase by over 55% annually. The latest estimates show that in 2020, 905,700 people worldwide were diagnosed with cancer, and 830,200 died from liver cancer [3]. Assuming that the current incidence rate and mortality rates remain unchanged, scientists evaluate that 1.4 million people may be diagnosed with cancer and 1.3 million people may die of liver cancer by 2040 [3].

Epidemiological data show that risk factors, including viral infections (Hepatitis B Virus (HBV) and Hepatitis C Virus (HCV)), metabolic changes (Alcoholic Steatohepatitis (ASH) and Non-Alcoholic Steatohepatitis (NASH)), chronic toxin exposure (such as aflatoxin), or parasitic infections (such as flukes), are crucial for the occurrence and development of most liver cancers [4]. The conventional treatment for liver cancer includes surgery, chemotherapy, and radiotherapy [5]. Due to the decrease in the liver’s regeneration ability, surgical resection is only suitable for early patients who usually do not have cirrhosis. In addition, surgical resection also carries the risk of postoperative complications. The treatment strategy for advanced liver cancer patients mainly relies on radiotherapy and the use of chemotherapy drugs. The long-term use of chemotherapy drugs (such as sorafenib) not only leads to significant drug resistance within six months [6] but also causes other issues, such as toxicity and/or drug ineffectiveness. Due to their weak efficacy and extreme systemic toxicity, these traditional therapies cannot significantly improve the prognosis of liver cancer. Further research is necessary to find new natural compounds that are found in food and have medicinal properties with the ability to reduce cancer recurrence rates and mortality rates.

The perennial herbaceous plant licorice is a widely used traditional Chinese medicine and is distributed in the temperate zones of Asia, represented by China. It has a variety of pharmacological activities, such as anti-diabetic, anti-asthmatic, antioxidant, anti-inflammatory, and anticancer effects [7,8,9,10,11,12]. Its pharmacological effects are related to the flavonoids it contains, among which licochalcones have considerable anticancer activity [13]. According to previous research, LCB caused HepG2 cell toxicity after 24 h by controlling the cell cycle during the G2/M phase and inducing cell apoptosis and intracellular ROS production [12]. Jun Wang et al. studied the anticancer mechanism of LCB based on miRNA and mRNA transcriptomics through high-throughput sequencing technology [14].

Licochalcones are a group of structurally similar licorice flavonoids and their derivatives, including LCA, LCB, LCC, LCD, LCE, LCF, and LCG. This study reveals the potential mechanisms by which licochalcones inhibit liver cancer through network pharmacology methods. Other structurally similar components in licorice were analyzed through machine-learning clustering, and the structural commonalities of these components related to liver cancer were found through quantitative calculations. Key residues between small components and key target proteins were also identified. In summary, we provide valuable insights into the molecular mechanisms of the anticancer effects of licorice flavonoids, providing new ideas for the design of small molecules for liver cancer drugs and serving as a reference for improving liver cancer treatment strategies.

## 2. Results

**Figure 1:**
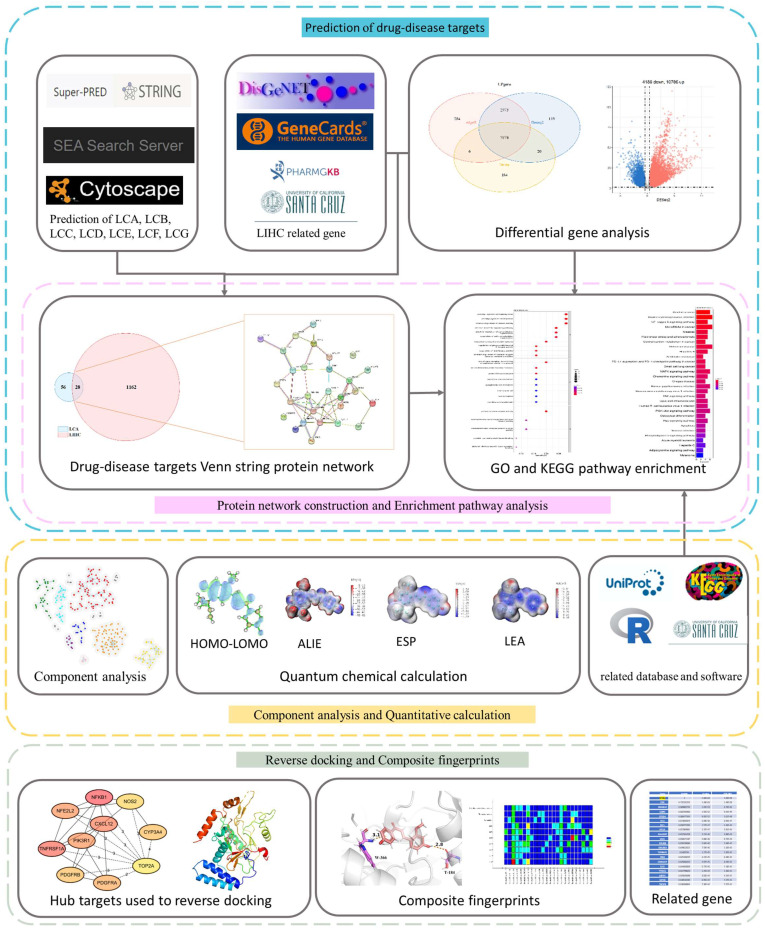
Workflow of network pharmacology combined with machine learning and quantitative calculation methods to identify the key targets and molecular mechanisms related to intervention with licochalcones in treating liver hepatocellular carcinoma (LIHC). It includes database screening, GO and KEGG pathway enrichment analyses, licorice component clustering analysis, PPI network mapping, quantum chemical calculation, target gene identification, complex fingerprint analysis, and related gene analysis.

Taking LCA as an example, the workflow of network pharmacology combined with machine-learning methods used to study the anticancer-related mechanisms of licorice flavonoids is shown in Figure 1. Firstly, we obtained and compared gene data between licorice flavonoids and liver cancer from different online databases, such as UCSC-TCGA, DisGeNET [15], GeneCards [16], Super-Pred [17], etc. Then, based on cross-gene data, GO and KEGG enrichment analysis was conducted to reveal the potential significance of licorice flavonoid molecules in liver cancer, including potential biological processes, pathways, and mechanisms. A cluster analysis of licorice components was conducted through unsupervised learning to identify potentially active molecules in licorice with similar efficacy. Quantitative calculations and analysis were conducted on licorice flavonoids using Gaussian 09, and their structural characteristics were explored. In addition, Discovery Studio was used for molecular docking between the main target protein, which was identified as nitric oxide synthase 2 (NOS2), and ten active ingredients.

### 2.1. Potential Targets for Liver Cancer and Licochalcones

**Figure 2:**
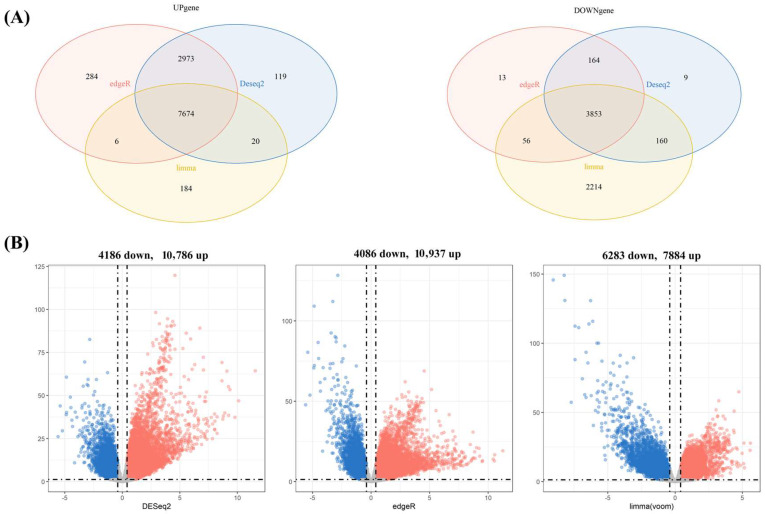
Differential gene analysis of liver cancer genes in the UCSC-TCGA database was performed using edgeR, Deseq2, and limma methods. The results show a total of 7674 upregulated genes and 3853 downregulated genes. (**A**) Three differential analysis methods for analyzing upregulation and downregulation genes in Venn plots. (**B**) Use volcano plots to display the upregulation and downregulation genes of three different differential analyses (the blue dots represent downregulated genes, while the red dots represent upregulated genes).

**Figure 3:**
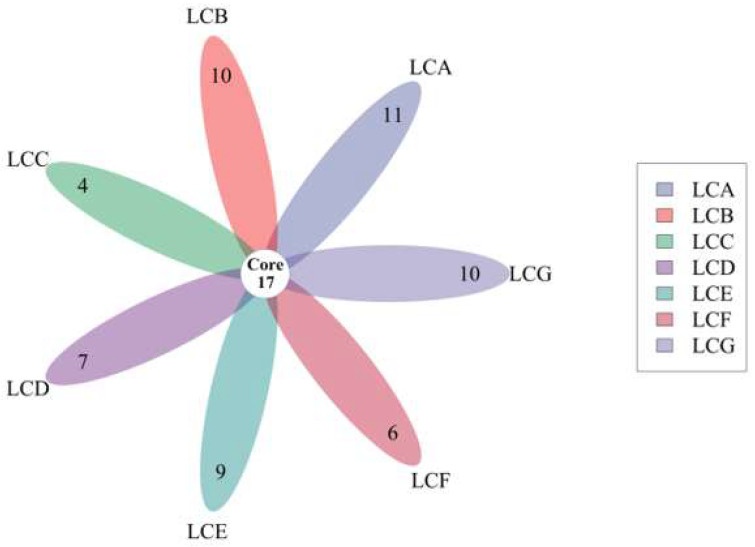
The petal diagram shows the cross genes that may significantly affect LIHC among seven licochalcones.

**Figure 4:**
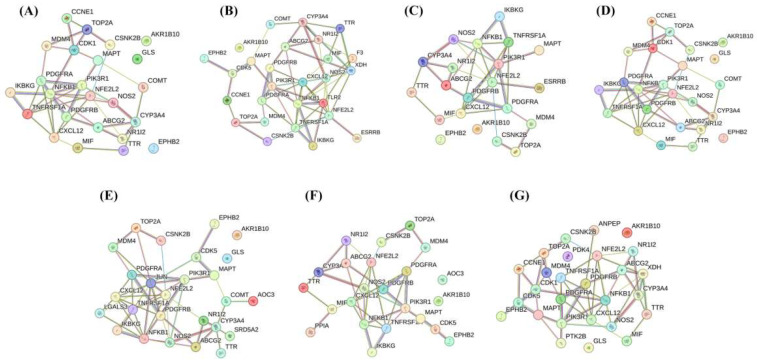
STRING database was used to perform PPI network analysis and display the interactions of the cross genes found in licochalcones and LIHC. (**A**–**G**): PPI network of LCA-LCG.

We obtained 2389 pathogenic genes/targets related to liver cancer from the DisGeNET [15], PharmGKB [16], and GeneCards [17] databases. By conducting differential gene analysis on the UCSC-TCGA database, a total of 7674 upregulated genes and 3853 downregulated genes were identified (Figure 2). A total of 1190 potential target genes for hepatocellular carcinoma were predicted to intersect with differentially expressed genes. In addition, regarding the 85, 109, 84, 91, 83, 72, and 82 pharmacological genes/targets of LCA, LCB, LCC, LCD, LCE, LCF, and LCG initially detected, Venn plots show that 28, 27, 21, 24, 26, 23, and 27 targets of these licochalcones molecules were significantly associated with liver cancer (Figure S1), and a petal diagram shows the same genes that may significantly affect LIHC among the seven licochalcones (Figure 3). The PPI network, including common targets, is shown in Figure 4.

### 2.2. GO and KEGG Pathway Enrichment Analysis

**Figure 5:**
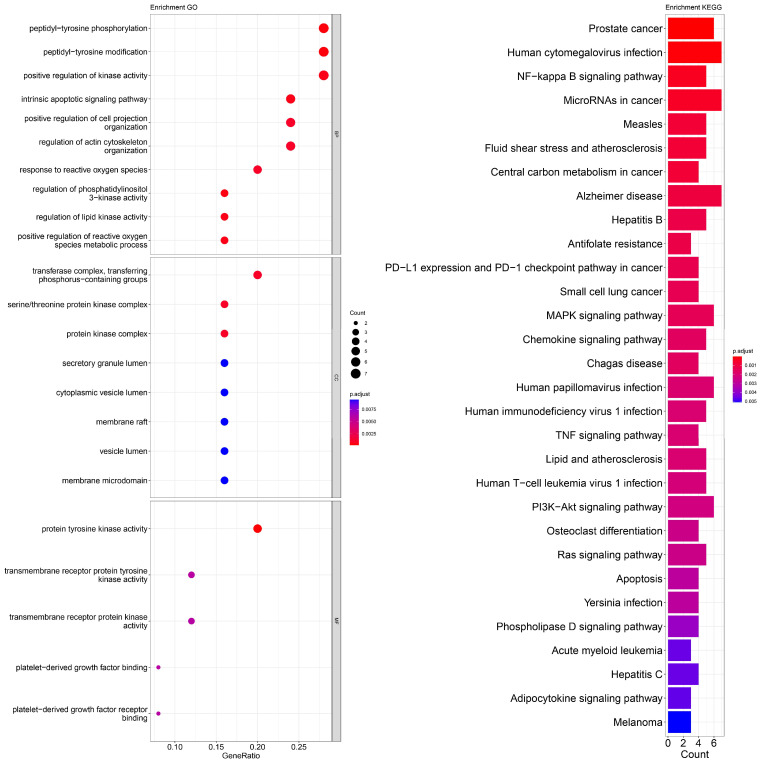
Visualization of GO and KEGG pathway enrichment analysis results. The adjusted *p*-value is indicated by the color of the bar, and the bubble size represents the number of genes. GO analysis is based on the genes common to LCA and LIHC, including those related to biological processes, molecular functions, and cellular components. KEGG pathway enrichment analysis emphasizes enriched pathways.

**Figure 6:**
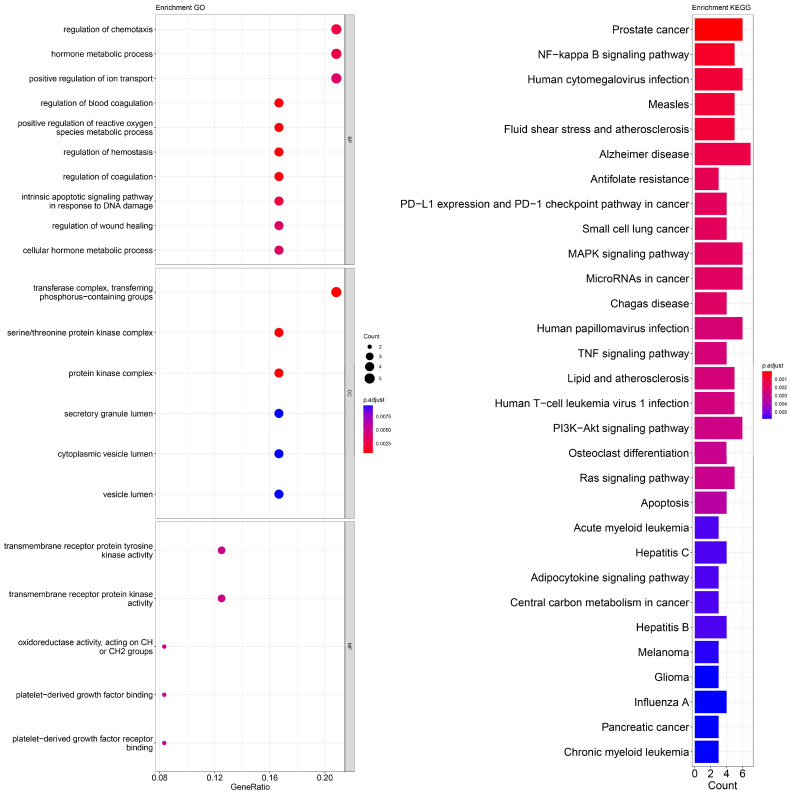
Visualization of GO and KEGG pathway enrichment analysis results. The adjusted *p*-value is indicated by the color of the bar, and the bubble size represents the number of genes. GO analysis is based on the genes common to LCB and LIHC, including those related to biological processes, molecular functions, and cellular components. KEGG pathway enrichment analysis emphasizes enriched pathways.

**Figure 7:**
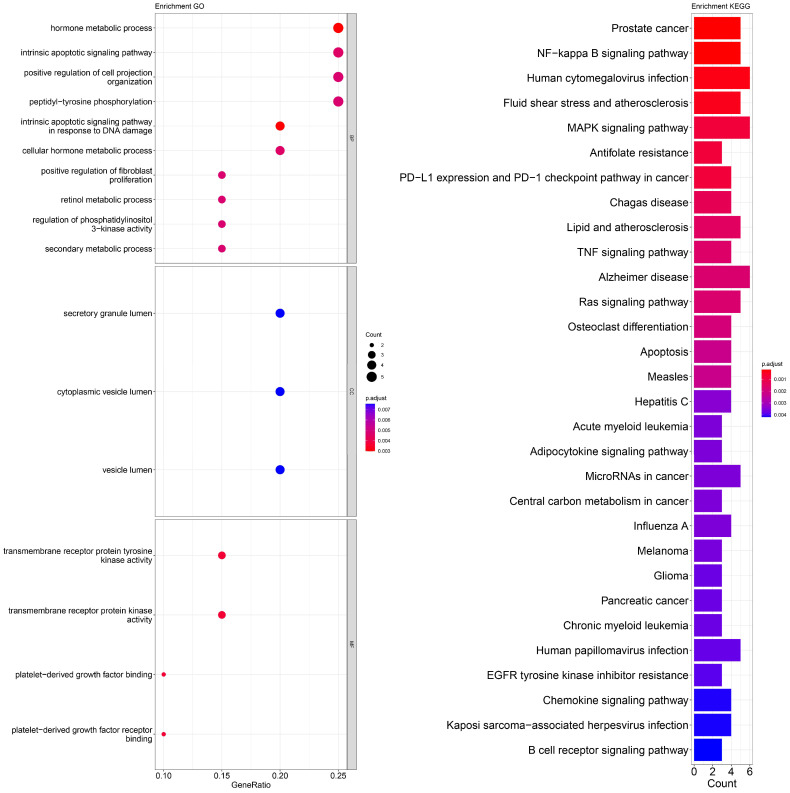
Visualization of GO and KEGG pathway enrichment analysis results. The adjusted *p*-value is indicated by the color of the bar, and the bubble size represents the number of genes. GO analysis is based on the genes common to LCC and LIHC, including those related to biological processes, molecular functions, and cellular components. KEGG pathway enrichment analysis emphasizes enriched pathways.

**Figure 8:**
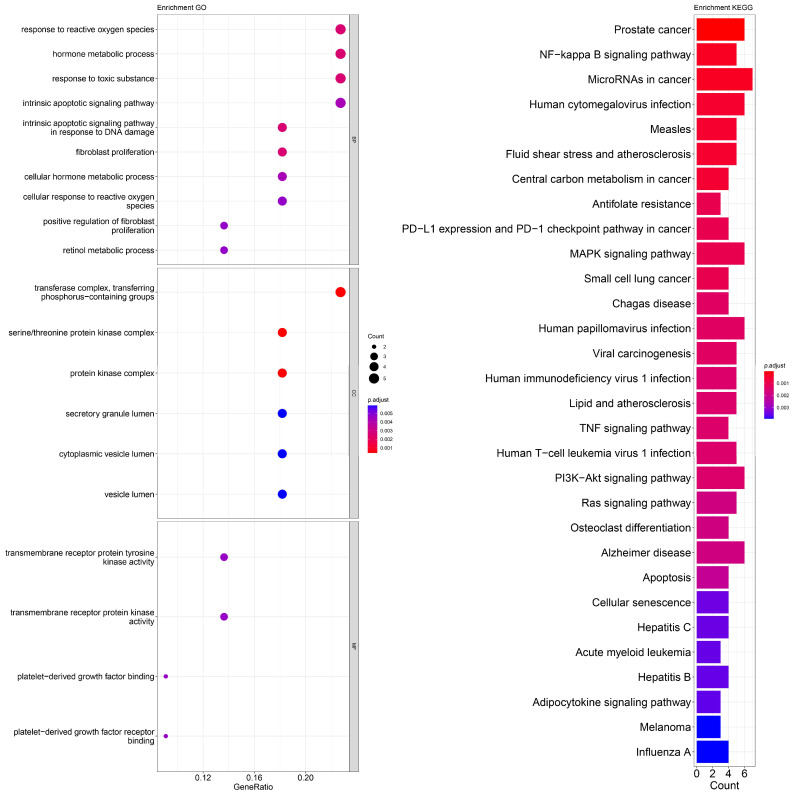
Visualization of GO and KEGG pathway enrichment analysis results. The adjusted *p*-value is indicated by the color of the bar, and the bubble size represents the number of genes. GO analysis is based on the genes common to LCD and LIHC, including those related to biological processes, molecular functions, and cellular components. KEGG pathway enrichment analysis emphasizes enriched pathways.

**Figure 9:**
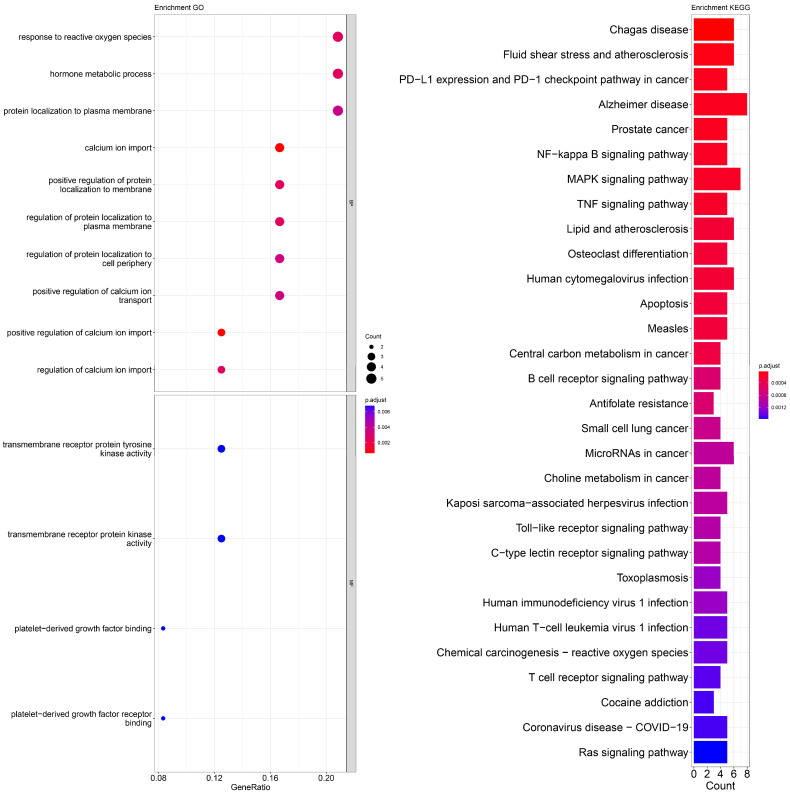
Visualization of GO and KEGG pathway enrichment analysis results. The adjusted *p*-value is indicated by the color of the bar, and the bubble size represents the number of genes. GO analysis is based on the genes common to LCE and LIHC, including those related to biological processes, molecular functions, and cellular components. KEGG pathway enrichment analysis emphasizes enriched pathways.

**Figure 10:**
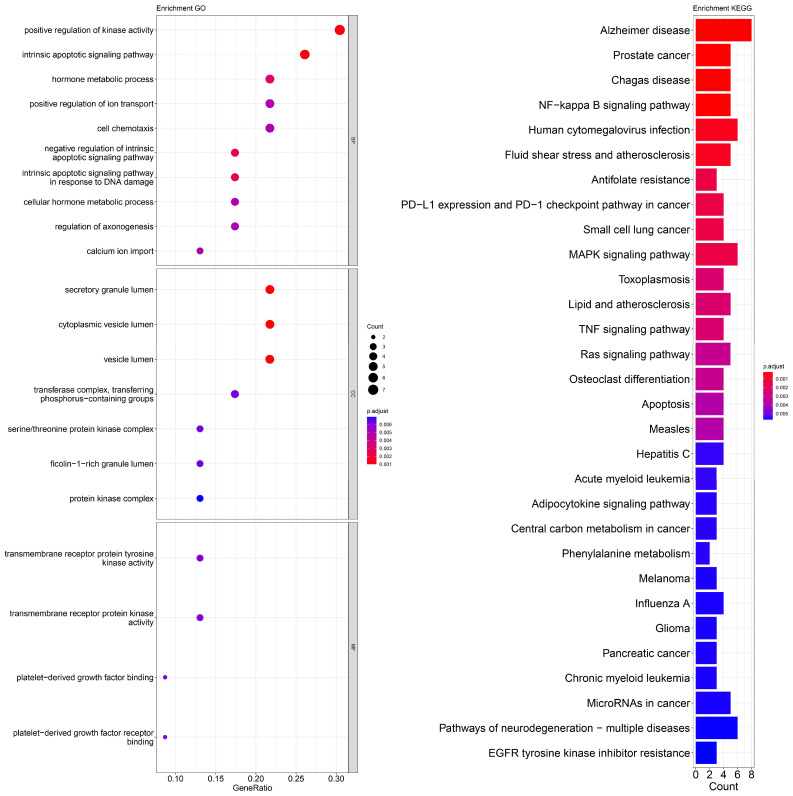
Visualization of GO and KEGG pathway enrichment analysis results. The adjusted *p*-value is implied by the color of the bar, and the bubble size represents the number of genes. GO analysis based on the genes common to LCF and LIHC, including those related to biological processes, molecular functions, and cellular components. KEGG pathway enrichment analysis emphasizes enriched pathways.

**Figure 11:**
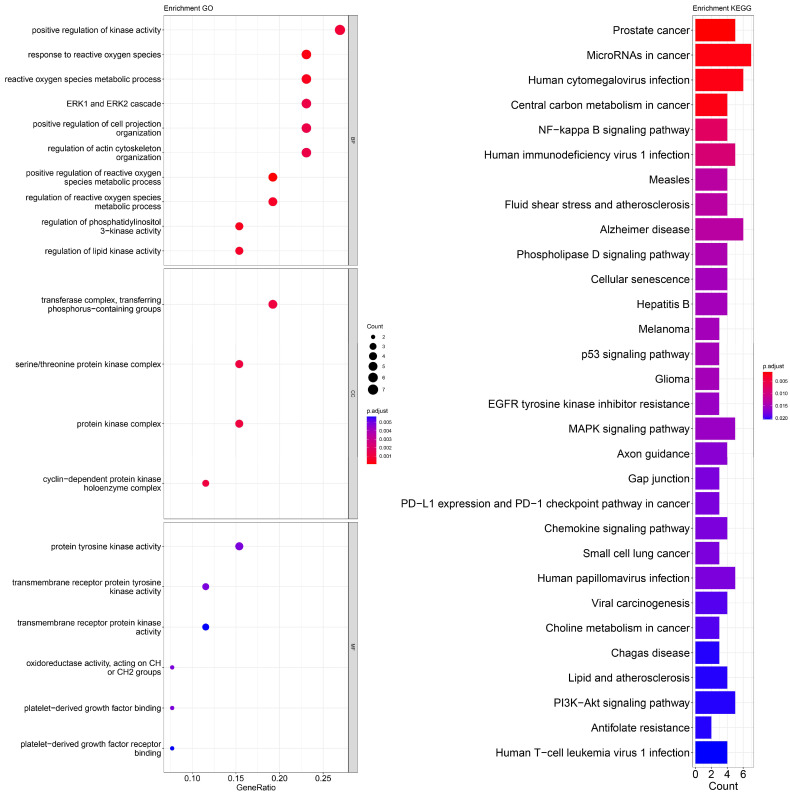
Visualization of GO and KEGG pathway enrichment analysis results. The adjusted *p*-value is indicated by the color of the bar, and the bubble size represents the number of genes. GO analysis is based on the genes common to LCG and LIHC, including those related to biological processes, molecular functions, and cellular components. KEGG pathway enrichment analysis emphasizes enriched pathways.

An enrichment analysis of GO and KEGG pathways was performed using cross genes related to liver cancer and licorice flavonoid small molecules. The calculated data are displayed as bubble plots and histograms obtained from GO and KEGG (Figure 5, Figure 6, Figure 7, Figure 8, Figure 9, Figure 10 and Figure 11).

Among potential biological processes related to the core targets, LCA, LCF, and LCG were all associated with the positive regulation of kinase activity; LCA, LCB, LCC, LCD, and LCF were all related to the intrinsic apoptotic signaling pathway; LCA, LCD, LCE, and LCG were all related to response to reactive oxygen species; and LCB, LCC, LCD, LCE, and LCF were all related to the hormone metabolic process. Among those enriched in cellular components, LCA, LCB, LCD, and LCG were all associated with the transfer complex and serine/threonine protein kinase complex; LCA, LCB, and LCG were all related to transferring phosphorus-containing groups; and LCC and LCF were both related to secretory granule lumen, cytoplasmic vessel lumen, and vessel lumen. LCA, LCB, LCC, LCD, LCE, LCF, and LCG were highly similar in molecular function, with all of them related to transmembrane receptor protein tyrosine kinase activity and transmembrane receptor protein kinase activity; LCC, LCD, LCE, and LCF were related to platelet-derived growth factor binding and platelet-derived growth factor receiver binding (Figure 5, Figure 6, Figure 7, Figure 8, Figure 9, Figure 10 and Figure 11).

The enriched KEGG pathways of LCA, LCB, LCC, LCD, LCE, LCF, and LCG were also highly similar, all containing pathways such as MicroRNAs in cancer, human cytomegalovirus infection, MAPK signaling pathway, NF-kappa B signaling pathway, and PD-L1 expression and PD-1 checkpoint pathway in cancer. Although there were differences in enrichment levels, they were all within a relatively high range (Figure 5, Figure 6, Figure 7, Figure 8, Figure 9, Figure 10 and Figure 11).

In addition, the unique potential biological processes of LCA included peptidyl-tyrosine modification, regulation of actin cytoskeleton organization, etc.; LCB’s unique potential biological processes included regulation of chemotaxis, positive regulation of ion transport, regulation of blood coagulation, etc.; LCD’s unique processes included response to toxic substance; and LCE’s unique processes included protein localization to plasma membrane and calcium ion import (Figure 5, Figure 6, Figure 7, Figure 8, Figure 9, Figure 10 and Figure 11).

### 2.3. Cluster Analysis of Licorice Components

**Figure 12:**
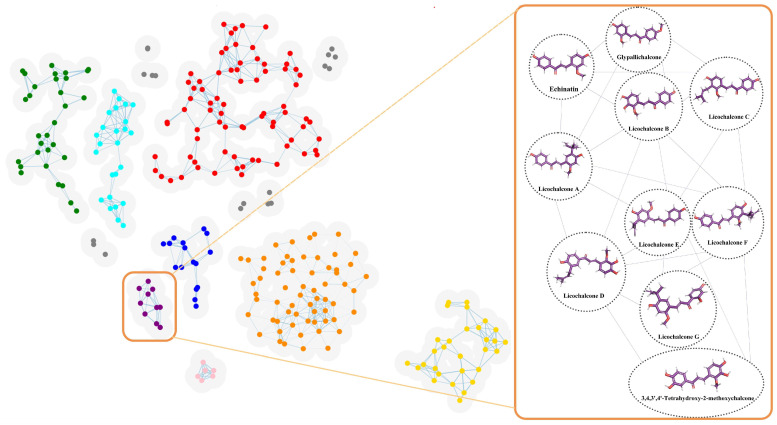
Cluster analysis of licorice components. Cluster analysis was conducted on the active ingredients in licorice using machine-learning methods. The results show that seven licochalcones, namely, LCA, LCB, LCC, LCD, LCE, LCF, and LCG, all belong to the same category. In addition, this category also includes Glyphallichalcone, Echinatin, and 3,4,3′,4′-Tetrahydroxy-2-methoxychalcone. Different colors represent different categories (Table S1).

The SMILES formulas of active small molecules obtained by searching for licorice components in the TCMSP database are shown in Table S1. The results of machine-learning clustering analysis show that the molecules LCA, LCB, LCC, LCD, LCE, LCF, and LCG of licorice flavonoids are all in the same category (purple part of Figure 12). In addition, this class also includes Glypallichalcone, Echinatin, and 3,4,3′,4′-Tetrahydroxy-2-methoxychalcone (Table 1).

### 2.4. Quantum Chemical Calculation of Ten Licorice Flavonoids

**Figure 13:**
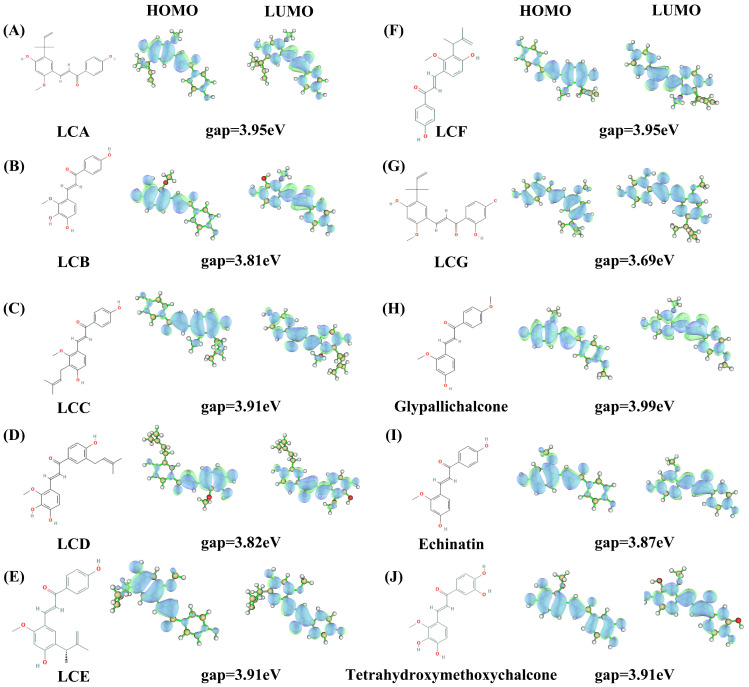
Molecular structures and HOMO–LUMO orbital diagrams of ten licorice components. (**A**) LCA, (**B**) LCB, (**C**) LCC, (**D**) LCD, (**E**) LCE, (**F**) LCF, (**G**) LCG, (**H**) Glypallichalcone, (**I**) Echinatin, (**J**) 3,4,3′,4′-Tetrahydroxy-2-methoxychalcone.

**Figure 14:**
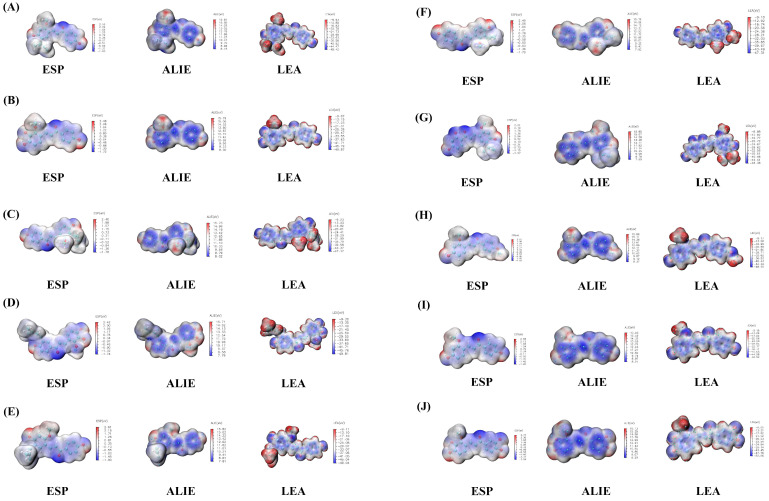
The ESP diagrams, LEA diagrams, and ALIE diagrams of ten licorice components. (**A**) LCA, (**B**) LCB, (**C**) LCC, (**D**) LCD, (**E**) LCE, (**F**) LCF, (**G**) LCG, (**H**) Glypallichalcone, (**I**) Echinatin, (**J**) 3,4,3′,4′-Tetrahydroxy-2-methoxychalcone.

Gaussian quantification calculations for the ten licorice flavonoids were performed using the B3LYP/6–31G* method. Firstly, the HOMO–LUMO orbit results (Figure 13) were as follows: (1) LCA: the energy gap between the HOMO orbit and LUMO orbit is 3.95 eV (the energy calculation results for HOMO and LUMO are −5.72 eV and −1.77 eV); (2) LCB: the energy gap between the HOMO orbit and LUMO orbit is 3.81 eV (the energy calculation results for HOMO and LUMO are −5.67 eV and −1.86 eV); (3) LCC: the energy gap between the HOMO orbit and LUMO orbit is 3.91 eV (the energy calculation results for HOMO and LUMO are −5.67 eV and −1.76 eV); (4) LCD: the energy gap between the HOMO orbit and LUMO orbit is 3.82 eV (the energy calculation results for HOMO and LUMO are −5.63 eV and −1.82 eV); (5) LCE: the energy gap between the HOMO orbit and LUMO orbit is 3.91 eV (the energy calculation results for HOMO and LUMO are −5.50 eV and −1.59 eV); (6) LCF: the energy gap between the HOMO orbit and LUMO orbit is 3.95 eV (the energy calculation results for HOMO and LUMO are −5.76 eV and −1.81 eV); (7) LCG: the energy gap between the HOMO orbit and LUMO orbit is 3.69 eV (the energy calculation results for HOMO and LUMO are −5.54 eV and −1.85 eV); (8) Glypallichalcone: the energy gap between the HOMO orbit and LUMO orbit is 3.99 eV (the energy calculation results for HOMO and LUMO are −5.75 eV and −1.77 eV); (9) Echinatin: the energy gap between the HOMO orbit and LUMO orbit is 3.87 eV (the energy calculation results for HOMO and LUMO are −5.54 eV and −1.67 eV); (10) 3,4,3′,4′-Tetrahydroxy-2-methoxychalcone: the energy gap between the HOMO orbit and LUMO orbit is 3.91 eV (the energy calculation results for HOMO and LUMO are −5.64 eV and −1.73 eV). Next, the electrostatic potential (ESP), local electron affinity energy (LEA), and average local ionization energy (ALIE) diagrams (Figure 14) of the Gaussian calculations demonstrate that the ten types of flavonoid small molecules have similar flavonoid structures, with two benzene rings and three carbons, which may be the functional structure of these compounds.

### 2.5. Hub Target Collection of Flavonoid Targets for Anti-Hepatoma Effects

**Figure 15:**
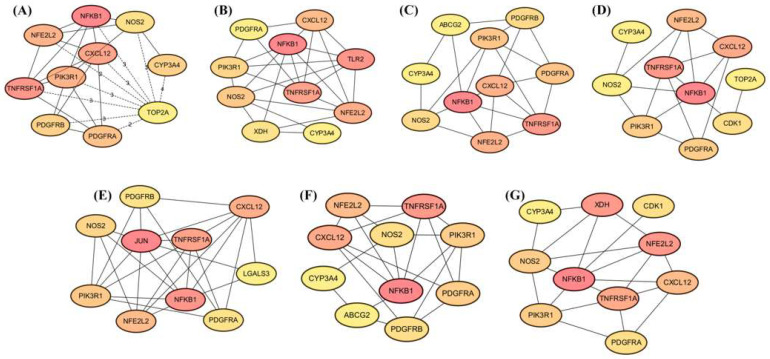
Protein interactions related to the anticancer activity of licochalcones. (**A**–**G**): The interactions of key targets of LCA-LCG. The interactions of hub targets related to intervention with licochalcones for treating LIHC were displayed using cytoHubba.

Using CytoHubba, a functional protein network of the flavonoid–liver-cancer system was created by applying the topological analysis method of Maximum Climate Centrality (MCC) [18]. The MCC algorithm was used to obtain scores for estimating the relationships between nodes and edges, with a higher score and darker color indicating a more significant correlation between genes and liver cancer. Then, we screened out the top 10 target genes with the highest scores for each active compound in the union set. The important proteins detected include ABCG2, CDK1, CXCL12, CYP3A4, JUN, LGALS3, NFE2L2, NFKB1, NOS2, PDGFRA, PDGFRB, PIK3R1, TLR2, TNFRSF1A, TOP2A, and XDH (Figure 15).

### 2.6. Binding of Ten Flavonoids to NOS2

**Figure 16:**
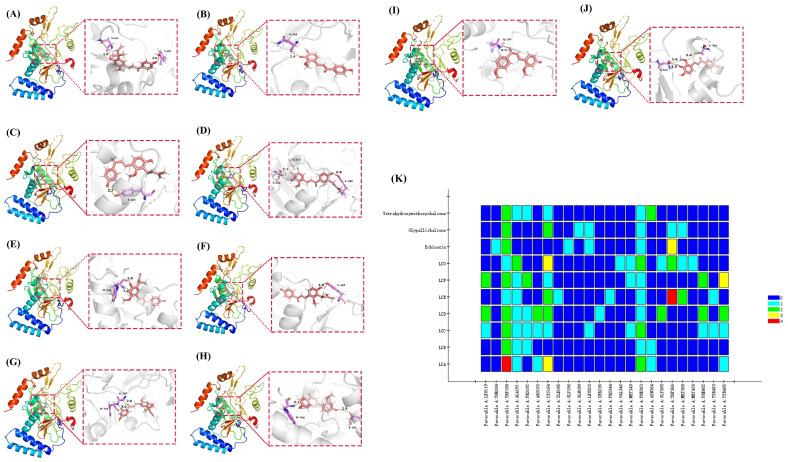
Complex fingerprint analysis of licorice components (**K**) and molecular docking indicated possible binding sites between LIHC and LCA (**A**), LCB (**B**), LCC (**C**), LCD (**D**), LCE (**E**), LCF (**F**), LCG (**G**), Glypallichalcone (**H**), Echinatin (**I**), and 3,4,3′,4′-Tetrahydroxy-2-methoxychalcone (**J**).

Using Echinatin, a representative flavonoid compound in the licorice component clustering analysis, 16 hub union target genes were reverse-docked to find the key target protein, and NOS2 received the highest score. Then, the NOS2 crystal structure was determined with PDBID 1dd7. Ten flavonoid compounds (LCA, LCB, LCC, LCD, LCE, LCF, LCG, Glypallichalcone, Echinatin, and 3,4,3′,4′-Tetrahydroxy-2-methoxychalcone) were used to analyze the complex fingerprint of NOS2 (Figure 16). Due to the lack of an effective small-molecule-binding crystal structure for NOS2, we selected binding sites based on the receptor cavity in Discovery Studio. In NOS2 (1DD7), we set the input site sphere parameters to 69.43719, −11.18631, 55.94179 (coordinates of the sphere), accompanied by 2.25 Å RMSD in the original protein. The results are as follows: the conventional hydrogen bonds between LCA and amino acids involve ASN364 and TYR485; the conventional hydrogen bond between LCB and amino acids involves ASN364; the conventional hydrogen bond between LCC and amino acids involves TYR483; the conventional hydrogen bonds between LCD and amino acids involve SER236 and TYR485, and the carbon–hydrogen bond between them is GLY365; the conventional hydrogen bond between LCE and amino acids involves TRP366; the conventional hydrogen bond between LCF and amino acids involves TYR485; the carbon–hydrogen bonds between LCG and amino acids involve GLY365 and TRP366; the conventional hydrogen bond between Glypallichalcone and amino acids involves GLN199; the conventional hydrogen bond between Echinatin and amino acids involves THR184, and the carbon–hydrogen bond between them involves TRP366; and the conventional hydrogen bonds between 3,4,3′,4′Tetrahydroxy-2-methoxychalcone and amino acids involves ASN364 and CYS194, and the carbon–hydrogen bond between them involves ASN364. According to compound fingerprint analysis, the common key residues of small molecules in ten flavonoids are TRP288 and PHE363. The research results indicate that these residues may be potential key residues for the action of small molecules of licorice flavonoids on liver cancer.

### 2.7. Analysis of NOS2-Related Genes

Linkedomics was used to select RNAseq data from the TCGA-LIHC database for the analysis of genes related to NOS2, and the results were screened according to the Pearson correlation coefficient (Table S2). According to Table 2, the genes most related to NOS2 include TIE1, CDH5, FLT4, PLEKHG1, ERG, ESAM, TSPAN18, NOTCH4, AOC3, TMEM204, RHOJ, BCL6B, MMRN2, CLEC14A, etc.

## 3. Discussion

In this study, network pharmacology analysis combined with machine learning and quantitative calculations revealed the biological processes, pathways, and mechanisms of licorice flavonoid molecules’ anti-hepatoma effects, expanding the range of potentially active small molecules in licorice that may have anticancer activity. The results of target screening and reverse docking indicate that the core cross-target is NOS2. The complex fingerprint and quantitative analysis of ten licorice flavonoids revealed the binding activity of the flavonoids with NOS2 in liver cancer, indicating that LCA, LCB, LCC, LCD, LCE, LCF, LCG, Glypallichalcone, Echinatin, and 3,4,3′,4′Tetrahydroxy-2-methoxychalcone can interact with specific proteins in liver cancer patients through key amino acid residues.

Nitric oxide (NO) is a reactive free radical that acts as a biological regulator in various processes. Its functional roles in tumors are complex and diverse, and it basically involves all of the characteristics of cancer, such as cell cycle progression, survival, apoptosis resistance, proliferation, metastasis, angiogenesis, or chemotherapy/radiotherapy resistance. It has neurotransmission, antibacterial, and anti-tumor activities [19]. In addition, NO affects tumors not only by regulating blood flow and maintaining vascular tension in tumor microvessels but also by acting as a cytotoxic molecule against tumors, with the ability to induce cell apoptosis and necrosis. Macrophages stimulated by pro-inflammatory factors upregulate inducible NOS2 and produce high steady-state NO concentrations. NO causes tumor cell death by inducing cell apoptosis and/or necrosis [20]. There are three levels of tumor–immune interactions. Firstly, chronic inflammation is beneficial for malignant transformation [21]; secondly, the development of tumors is influenced by tumor rejection or tumor dormancy after being controlled by the immune system [22,23,24]; and thirdly, tumors contain immune cell populations that support tumor development, rather than inducing tumor destruction [25]. Each of the three levels of tumor–immune interactions mentioned above is influenced by NOS2, and the NO produced by NOS2 during chronic inflammation may cause genetic toxicity, supporting malignant transformation [26]. NO explosions produced by macrophages may kill transformed cells [27], and low levels of NO produced by myelocytes may inhibit tumor-killing lymphocytes [25].

Many clinical trials have shown that expression level differences in NOS2 exist in more than 50% of patients with glioma, breast cancer, prostate cancer, pancreatic cancer, melanoma, liver cancer, cervical cancer, ovarian cancer, nasopharyngeal cancer, lung cancer, stomach cancer, colon cancer, and esophageal cancer [28]. More importantly, NOS2 has been proven to predict a poor prognosis in breast cancer, glioma, melanoma, pancreatic cancer, gastric cancer, liver cancer, and colon cancer. In many cases, it is associated with an increase in blood vessels and metastatic potential [28]. In addition, studies have shown that LCA, LCB, and LCD significantly inhibit NO production induced by LPS and the expression of TNF and αMCP-1 in RAW264.7 cells and primary macrophages [29,30].

Many in vitro studies have focused on the antiproliferative and proapoptotic effects of NO donors on hepatocellular carcinoma (HCC) cell lines, as well as the synergistic killing of tumor cells by NO and sorafenib in vitro [31]. Research has shown that the concentration of NO-derived products in the plasma of HCC patients increases [32], and the expression of NOS2 in HCC is lower compared to normal liver tissue [33]. NOS2 is positively correlated with tumor proliferation and microvascular formation, negatively correlated with cell apoptosis, and significantly correlated with a poor prognosis in HCC [34]. In liver cancer cells, the overexpression of NOS2 and NOS3 induces a transition between apoptosis and autophagy by disrupting the Beclin 1/Vps34 association and increasing the Bcl-2/Beclin 1 interaction [32]. The expression of NOS2 in CD24 + CD133 + liver CSC promotes NO/cGMP/PKG, driving Notch signaling and stemness characteristics in vitro and in vivo, and accelerates HCC initiation and tumor formation in the murine HCC tumor [35]. HCC often occurs in patients with HBV or HCV. The X gene of HBV (HBx) is associated with the development of HBV-related HCC induced by NOS2 [36]. HBx-induced NOS is associated with NF-kB signaling [37].

In terms of GO and KEGG pathway enrichment, the enrichment pathways of LCA, LCB, LCC, LCD, LCE, LCF, and LCG are highly similar, all containing pathways such as MicroRNAs in cancer, human cytomegalovirus infection, MAPK signaling pathway, NF-kappa B signaling pathway, and PD-L1 expression and PD-1 checkpoint pathway in cancer. The gene commonalities among licochalcones affecting the intersection targets in liver cancer include 17 genes, including CXCL12, CYP3A4, MAPT, MDM4, NFKB1, NOS2, PIK3R1, TOP2A, etc., which are also concentrated in the above enrichment pathways.

In HCC, miRNAs can serve as carcinogenic genes, promoting the progression of liver cells toward HCC, or as tumor inhibitors to prevent this process [38]. An increase in carcinogenic miRNA levels leads to a decrease in the translation of its gene targets, which contributes to the development and progression of HCC. On the contrary, miRNAs can also act as tumor inhibitors to prevent the expression of their oncogenic targets, so the downregulation of these miRNAs allows for more expression of these oncogenic genes, promoting the development and progression of HCC. The development from normal liver cells to HCC is a multi-stage process. Some changes are partially mediated by miRNA expression profiles, including liver fibrosis and liver regeneration mediated by hepatic stellate cells [39]. Cytomegalovirus infection is the most common viral infection after liver transplantation for hepatoblastoma, and research on nonmalignant diseases has shown an incidence of 30–80% [40].

The activity of PI3K is related to pathological cell growth and tumorigenesis. At present, high-throughput sequencing methods have confirmed that genetic overactivation of PI3K/AKT signaling is considered one of the most common driving mechanisms in many cancers [41]. Cancer-promoting levels of NO were found at concentrations of 100 to 500 nM, which led to the activation of pathways involving RAS/ERK, PI3K/Akt/b-catenin, HIF-1α, NrF2, and TGFb. Unlike epidermal growth factor or other growth factors, the level of NO is a common driving factor in the carcinogenic pathway and is usually associated with a poor prognosis in patients. And NOS2 is the only subtype that can achieve these NO levels over a period of time, indicating that NOS2 may play a role in both tumor-promoting and anti-tumor processes [42]. However, the potential cytotoxic activity of macrophages is often compromised in the tumor microenvironment, where they instead exert tumor-promoting activity. The contributing factors are signals generated by surviving and dying tumor cells, the attraction and activation of bone-marrow-derived inhibitory cells, and hypoxia. The expanding tumor faces hypoxic areas. A limited oxygen supply not only affects the accumulation of hypoxia-inducible factors-1 and -2 (HIF-1α, HIF-2α) but also weakens the activity of NOS2. Therefore, the degree of hypoxia may not only affect the efficacy of anticancer drugs on tumor cells but also affect the formation of reactive nitrogen and reactive oxygen species (ROS) through hypoxia or the HIF transcription system [43,44,45]. The activation of the HIF system is closely related to the formation of NO and affects the expression of macrophage phenotype markers, thereby increasing tumor progression.

The HBx is the most common open reading framework integrated into the host genome in HCC. The integrated HBx often undergoes mutations and has a diminished ability to function as a transcriptional cotransactivator and to activate the NF-kappa B pathway [46]. The inhibition of IFN-γ induced by regulatory T cells (Tregs) can be partially blocked by specifically neutralizing PD-1 and PD-L1 antibodies in HCC patients. In HCC, peripheral Tregs upregulate checkpoint inhibitors and promote systemic immune dysfunction through several inhibitory pathways, which may promote the development of tumors at a young age. Blocking PD-L1/PD-1 interactions in vitro selectively interfered with inhibitory Treg–T-effector cell interactions in patients with HCC and resulted in improved antitumoral activity against checkpoint-inhibitor-negative tumor cells, as well [47].

In addition, the unique potential biological processes of LCA include peptidyl-tyrosine modification, regulation of actin cytoskeleton organization, etc. The YT521-B homology (YTH) domain family plays an important role in the development of HCC. The function of the YTH domain family is related to several cancer-related pathways, including peptide serine modification and negative regulation of cellular component movement [48].

The unique potential biological processes of LCB include the regulation of chemotaxis, regulation of blood coagulation, etc. Chemokines and their receptors play a crucial role in determining the metastasis destinations of tumor cells [49]. Coagulation factor VII (FVII) is synthesized physiologically in the liver and then released into the bloodstream. The binding of FVII to tissue factor (TF) is related to the metastatic potential of tumor cells [50]. Both LCB and LCD are related to ion transport. Liver cancer stem cells (LCSCs) mediate therapeutic resistance and are associated with adverse outcomes in patients with HCC. Fibroblast growth factor (FGF) −19 is an important oncogenic driver gene in HCC and is associated with a poor prognosis. Mechanistically, FGF19/FGFR4 signaling stimulates store-operated Ca entry (SOCE) through both the PLCγ and ERK1/2 pathways. Subsequently, SOCE calcineurin signaling promotes the activation and translocation of nuclear factor of activated T cells (NFAT) c2, which transcriptionally activates the expression of stem-related genes [51].

LCD pathways include the response to toxic substance. Per-/polyfluoroalkyl substances (PFASs) are widely present in human blood and have certain toxic effects on the liver. This new discovery supports the evidence of a positive correlation between PFAS exposure, changes in specific tumor markers, and the risk of liver cancer [52]. LCE pathways include protein localization to plasma membrane. The localization of proteins on the cell membrane is closely related to the metastasis of liver cancer [53].

According to the docking results, it can be seen that the key residue between LCA, LCB, 3,4,3′,4′Tetrahydroxy-2-methoxychalcone, and NOS2 is ASN364. The O-H on the first benzene ring of the three compounds forms a traditional hydrogen bond with the C=O double bond of ASN364; the –OCH3 on the first benzene ring of 3,4,3′,4′Tetrahydroxy-2-methoxychalcone forms a hydrocarbon bond with the C=O double bond of ASN364. The key residue between LCA, LCF, and NOS2 is TYR485. The O-H bond on the second benzene ring of LCA forms a traditional hydrogen bond with the O atom of TYR485; the O-H bond on the first benzene ring of LCF forms a traditional hydrogen bond with the O atom of TYR485. The key residue between LCE, LCG, Echinatin, and NOS2 is TRP366. The O-H on the first benzene ring of LCE forms a traditional hydrogen bond with the C=O double bond of TRP366, while the center of the first benzene ring of LCE forms a Pi-donor hydrogen bond with the N-H of TRP366; the second benzene ring center of LCG forms a Pi-donor hydrogen bond with the N-H of TRP366; and the second benzene ring center of Echinatin forms a Pi-donor hydrogen bond with the N-H of TRP366. The natural reversal of small molecules and changes in their dynamics during the docking process may be the reasons for the involvement of different parts of the molecule in key residue interactions.

From the HOMO-LUMO orbital diagrams of the ten flavonoids, it can be seen that the sites where orbital transitions are prone to occur are all concentrated on the skeleton, dominated by two benzene rings, which is consistent with the positions of the interactions between small molecules and target proteins that we have identified. In addition, the energy gap between HOMO and LUMO orbitals can reflect the binding activity of small molecules in the reaction. From Figure 13, it can be concluded that binding reactions with LCG are the easiest, while those with Gly are the most difficult. Based on the structural differences between the two molecules, it is speculated that an appropriate side chain on the benzene ring can increase the activity of small molecules.

On the molecular surface, the contribution of electrons and the contribution of the atomic nucleus can be counterbalanced, and the uneven distribution of the electron density can lead to positive and negative electrostatic potentials on the molecular surface. The process in which molecules approach each other through electrostatic attraction in the initial stage of the chemical reaction is closely related to the electrostatic effect generated by molecules. Therefore, we can predict which chemical reaction is most likely to occur at which site by analyzing the distribution of electrostatic potential on the van der Waals surfaces of molecules. It is generally believed that atoms with a negative (positive) electrostatic potential are more likely to undergo electrophilic (nucleophilic) reactions. According to the ESP, LEA, and ALIE plots, it can be seen that the benzene ring sites of the ten flavonoids all have relatively negative electrostatic potentials. The LEA plot shows that there is no bond formation at sites with obvious positive electrostatic potentials, indicating that the electrostatic potential at the NOS2 active site may be positive, leading to the complementary contact of small molecule benzene rings. In addition, the use of electrostatic potential can predict and explain the relative orientation of molecules in the complex. In the docking results, we found the involvement of different parts of the molecules in key residue interactions. The ESP, LEA, and ALIE plots can serve as evidence to support the differences in their relative orientations. For example, the O-H bond on the second benzene ring of LCA forms a traditional hydrogen bond with the O atom of TYR485; the O-H bond on the first benzene ring of LCF forms a traditional hydrogen bond with the O atom of TYR485. The difference in the bonding site may be due to the repulsion between the excess side chains of the first benzene ring of LCA and the electrostatic potential of the target protein, resulting in a change in orientation and the participation of the second benzene ring in the reaction.

The HOMO-LUMO orbital diagrams and ESP diagrams reflect the structural characteristics of small molecules, and the positions of different side chains of different small molecules may be the reason for the differences in their targets and pathways of action.

In summary, this study indicates that ten small molecules of licorice flavonoids mainly exhibit their anti-hepatoma relief effect through the positive regulation of kinases and response to reactive oxygen species. In addition, bioinformatics analysis showed that the active ingredients studied exert their effects through the NF-κB signaling pathway, central carbon metabolism in tumors, and the PDL1-PD1 checkpoint pathway in cancer. Finally, our analysis also revealed the potential of NOS2 as a potential biomarker for diagnosing liver cancer.

## 4. Materials and Methods

### 4.1. Prediction of Hepatocellular-Carcinoma-Related Targets and Licochalcone Targets

Firstly, potential target genes in hepatocellular carcinoma were screened in DisGeNET (https://www.disgenet.org/, accessed on 28 July 2023) [15], GeneCards (https://www.genecards.org/, accessed on 28 July 2023) [16], PharmGKB (https://www.pharmgkb.org/, accessed on 28 July 2023) [54], and UniProt (https://www.uniprot.org/, accessed on 28 July 2023) databases [55]. We identified 632 potential liver cancer target genes with scores >0.1 in DisGeNET, 2093 potential target genes with scores >5 in GeneCards, and 16 potential target genes in PharmaGKB. After deleting the repeated parts in the three databases, 2389 potential hepatocellular carcinoma target genes were identified. Secondly, 7674 upregulated genes and 3853 downregulated genes were identified by differential gene analysis using edgeR, Deseq2, and limma methods in the UCSC-TCGA database. Finally, intersecting the predicted potential target genes and differential genes resulted in a total of 1190 potential target genes for hepatocellular carcinoma.

Then, we used SEA (http://sea.bkslab.org/, accessed on 28 July 2023) [56] and Super-Pred [17] (https://prediction.charite.de/subpages/target_prediction.php, accessed on 28 July 2023) databases for the cross predictions of LCA, LCB, LCC, LCD, LCE, LCF, and LCG target genes based on active components and disease target genes, generating 28, 27, 21, 24, 26, 23, and 27 related cross genes, respectively.

### 4.2. Construction of Protein–Protein Interaction (PPI) Network

The PPI network was built using the STRING database (http://string-db.org/, accessed on 29 July 2023) [57] to evaluate the potential interactions between the screened hub targets. Subsequently, Cytoscape software (version 3.9.1; https://cytoscape.org/, accessed on 22 November 2022) was used [58]. Then, the topological characteristics of the PPI network were analyzed, and the top 10 hub genes were selected using the Cytoscape analysis tool.

### 4.3. GO and KEGG Enrichment Analysis

Based on the core target information, enrichment analysis was conducted on the Gene Ontology (GO) [59,60] biological processes and the Kyoto Encyclopedia of Genes and Genomes (KEGG) using R packages such as BiocManager, ClusterProfiler, AnnotationHub, org.Hs.eg, pathview, dplyr, and ggplot2. Using cut-off values of *p* = 0.01 and *q* = 0.01, we retrieved GO information from org.Hs.eg.Db of Bioconductor. The final results were visualized using bar and bubble charts.

### 4.4. Cluster Analysis of Licorice Components

RDKit was used to calculate Morgan fingerprint vectors with a length of 2048 and a molecular radius of 2. The similarity between fingerprints was calculated using Dice coefficients, and the formula is as follows:

$$DiceSimilarity\left(a,b\right)=\frac{2\times \left(a\cap b\right)}{a+b}$$

Here, *a* and *b* are the substructure characteristics of the two molecules. Then, we used *t*-distributed random neighbor embedding (*t*-SNE) to reduce the dimensionality to a two-dimensional space to obtain their relative spatial positions. In order to unify the number of digits of fingerprint vectors, the explicit bit vector method was used to generate ECFP fingerprints during the dimensionality reduction process; the number of bits was set to 1024, and the radius was 2. Then, we constructed a chemical spatial network based on the node coordinates and edge connections generated in the first two steps. This space represents the chemical space of the molecules. In this method, if the distance between two molecules is less than 1/24 of the distance between the farthest two molecules in the entire molecular set, the molecules are considered a group, and then all molecules are traversed to complete the clustering. The compound with the maximum closeness centrality will be the representative compound of this group.

Finally, we clustered and identified the representative compounds of each group. The grouping of compounds was determined based on whether there were edge connections between molecules and whether their positions were close. The average intragroup similarity (MWGS) was calculated for each group of molecules:

(1) $$MWGS\left(S_{i},n\right)=\frac{(\sum_{i=1}^{n}S_{i})-1}{n}$$

In the formula, *n* is the number of molecules in the group, and $S_{i}$ is the similarity between the molecule and the *i*-th molecule in the group.

The compound with the largest MWGS in this group is considered a representative compound of this group.

Clustering was based on Matplotlib’s chemical spatial network (https://matplotlib.org/ accessed on 19 August 2023). Visualization methods include visualizing the node radius to represent distance truncation and edge thickness to represent the Dice similarity coefficient between two molecules. Through machine learning, we identified other structurally similar active small molecules in licorice [61].

### 4.5. Cluster Analysis of Licorice Components

Ten flavonoid molecules were quantitatively calculated using Gaussian 09W and Gaussian View 5.0 [62]. The Gaussian calculation method was the B3LYP of the ground-state DFT, and we made the Basis Set 6–31G*. Multiwfn [63] and VMD [64] were used to plot visualization graphs for quantitative calculations, including HOMO-LUMO orbitals, electrostatic potential (ESP), average local ionization energy (ALIE), and local electron affinity (LEA).

### 4.6. Compound Fingerprint Analysis

Discovery Studio was used to evaluate the interactions between ten flavonoid small molecules and central targets. Based on the PPI analysis results (the union of the top 10 hub targets with high values), the most representative compound, Echinatin, in component enrichment analysis was used for reverse docking of the union. The crystal structure (1dd7, resolution: 2.25 Å) of NOS2 was selected as the receptor protein from the RCSB protein database (http://www.rcsb.org/pdb, accessed on 27 August 2023) [65] for further molecular docking. The filtering standard for selecting the reported protein was the organism ‘Homo’. Before docking, ligands and water were removed from the NOS2 crystal structure to prepare the NOS2 protein. Due to the lack of the crystal structure of NOS2 with active small molecules, the molecular docking sites were determined based on the acceptor cavity structure of NOS2. Then, the ten flavonoid molecules were used as ligands for docking. Before docking, the energy of all small molecules was minimized by preparing them and adding a CHARMM force field [66]. In addition, LibDock [67] was used for batch docking between the ten flavonoid molecules and NOS2. Using Discovery Studio [68] for complex fingerprint analysis, hydrogen bonding and coordination interactions between the receptor protein’s active-site residues and flavonoid small molecule postures were identified. Finally, Pymol software (version 2.3.4) [69] was used to display the details of interactions between flavonoid small molecule targets.

## 5. Conclusions

By enriching cross genes in liver cancer, we found that licorice flavonoids pass through the NF-κB signaling pathway, central carbon metabolism in cancer, and PDL1 expression and PD1 checkpoint pathway in cancer to exert their anti-hepatoma effects. In addition to LCA-G, there are also Glypallichalcone, Echinatin, and 3,4,3′,4′Tetrahydroxy-2-methoxychalcone in licorice, which have similar structures and may play similar roles. NOS2 is a key target protein shared by ten licorice flavonoid molecules, with ASN364, GLY365, TRP366, and TYR485 identified as key residues. In summary, using network pharmacology analysis, combined with machine learning and quantitative calculations, we comprehensively revealed the biological processes, targets, and molecular mechanisms of LCA, LCB, LCC, LCD, LCE, LCF, LCG, Glypallichalcone, Echinatin, and 3,4,3′,4′Tetrahydroxy-2-methoxychalcone in liver cancer, and our results indicate that they can serve as promising compounds for the treatment of liver cancer.

## Figures and Tables

**Table 1:** Cluster analysis results for licorice components (the category that includes licochalcones).

Molecule Name	Smiles	Color	Closeness Centrality
Glypallichalcone	COC1=CC=C(C=C1)C(=O)/C=C/C2=C(C=C(C=C2)O)OC	Purple	0.473
Echinatin	COC1=C(C=CC(=C1)O)/C=C/C(=O)C2=CC=C(C=C2)O	Purple	0.512
Licochalcone B	COC1=C(C=CC(=C1O)O)/C=C/C(=O)C2=CC=C(C=C2)O	Purple	0.501
Licochalcone C	CC(=CCC1=C(C=CC(=C1OC)/C=C/C(=O)C2=CC=C(C=C2)O)O)C	Purple	0.378
Licochalcone D	CC(=CCC1=C(C=CC(=C1)C(=O)/C=C/C2=C(C(=C(C=C2)O)O)OC)O)C	Purple	0.343
Licochalcone E	C[C@H](C1=C(C=C(C(=C1)/C=C/C(=O)C2=CC=C(C=C2)O)OC)O)C(=C)C	Purple	0.445
Licochalcone F	CC(C1=C(C=CC(=C1OC)/C=C/C(=O)C2=CC=C(C=C2)O)O)C(=C)C	Purple	0.464
Licochalcone G	CC(C)(C=C)C1=C(C=C(C(=C1)/C=C/C(=O)C2=C(C=C(C=C2)O)O)OC)O	Purple	0.308
3,4,3′,4′-Tetrahydroxy-2-methoxychalcone	COC1=C(C=CC(=C1O)O)/C=C/C(=O)C2=CC(=C(C=C2)O)O	Purple	0.438
Licochalcone A	CC(C)(C=C)C1=C(C=C(C(=C1)/C=C/C(=O)C2=CC=C(C=C2)O)OC)O	Purple	0.357

**Table 2:** Related analysis of NOS2-related genes.

Query	Statistic	*p*-Value	FDR (BH)
NOS2	1.000	1.00 × 10^−20^	1.00 × 10^−16^
TIE1	0.451	5.25 × 10^−20^	5.23 × 10^−16^
CDH5	0.441	4.05 × 10^−19^	2.69 × 10^−15^
FLT4	0.431	3.18 × 10^−18^	1.58 × 10^−14^
PLEKHG1	0.429	4.47 × 10^−18^	1.78 × 10^−14^
ERG	0.416	6.12 × 10^−17^	2.03 × 10^−13^
ESAM	0.412	1.25 × 10^−16^	3.55 × 10^−13^
TSPAN18	0.408	2.79 × 10^−16^	6.94 × 10^−13^
NOTCH4	0.406	3.55 × 10^−16^	7.28 × 10^−13^
AOC3	0.406	3.84 × 10^−16^	7.28 × 10^−13^
TMEM204	0.406	4.02 × 10^−16^	7.28 × 10^−13^
RHOJ	0.404	5.06 × 10^−16^	8.40 × 10^−13^
BCL6B	0.404	5.69 × 10^−16^	8.42 × 10^−13^
MMRN2	0.403	5.92 × 10^−16^	8.42 × 10^−13^
CLEC14A	0.403	6.35 × 10^−16^	8.44 × 10^−13^

## Data Availability

Not applicable.

## Supplementary Materials

The supporting information can be downloaded at https://www.mdpi.com/article/10.3390/ijms242115935/s1.
