# Supplementary material for: Network Pharmacology Combined with Machine Learning to Reveal the Action Mechanism of Licochalcone Intervention in Liver Cancer
Source: Int J Mol Sci. 2023 Nov 3;24(21):15935. doi: 10.3390/ijms242115935 (PMC10649909; doi:10.3390/ijms242115935)
Supplement: Supplementary file 1 [file ijms-24-15935-s001.zip › ijms-2652161-supplementary.pdf]

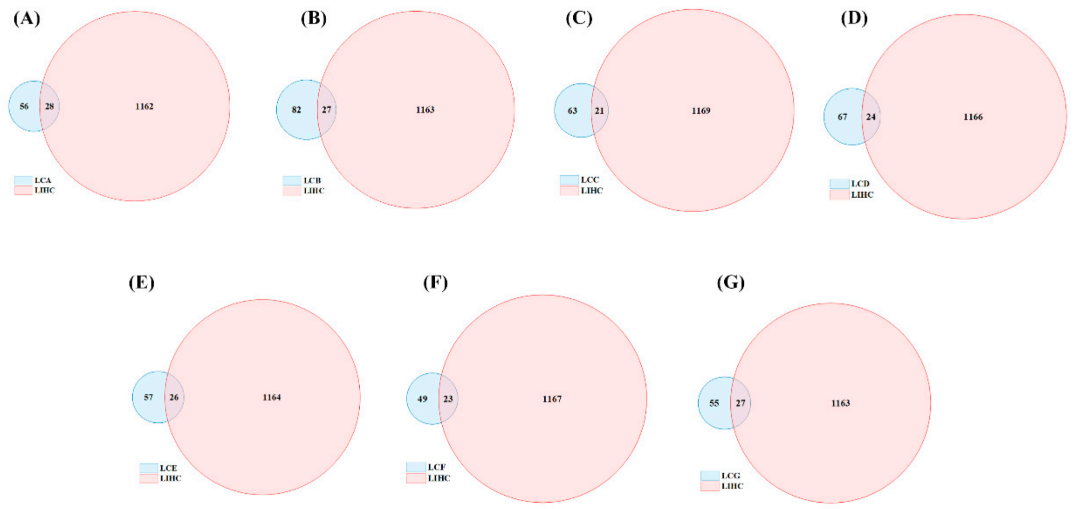

Figure S1. Venn diagrams showing the cross-genes shared between licochalcones and LIHC. (A) The number of common genes shared between LCA and LIHC is 28. (B) The number of common genes shared between LCB and LIHC is 27. (C) The number of common genes shared between LCC and LIHC is 21. (D) The number of common genes shared between LCD and LIHC is 24. (E) The number of common genes shared between LCE and LIHC is 26. (F) The number of common genes shared between LCF and LIHC is 23. (G) The number of common genes shared between LCG and LIHC is 27.

Table S1. Cluster analysis results of licorice components.

Molecule name	Smiles	Color	Closeness Centrality
protocatechuic acid	C1=CC(=C(C=C1C(=O)O)O)O	darkorange	0.114406586
o-xylene	CC1=CC=CC=C1C	darkorange	0.159598566
m-xylene	CC1=CC(=CC=C1)C	darkorange	0.153346397
p-xylene	CC1=CC=C(C=C1)C	darkorange	0.163053571
(L)-alpha-Terpineol	CC1=CC[C@H](CC1)C(C)(C)O	darkorange	0.155636748
Arachic acid	CCCCCCCCCCCCCCCCCCCC(=O)O	darkorange	0.142167105
α-cubebol	C[C@@H]1CC[C@H]([C@H]2[C@]13[C@@H]2[C@@](CC3)(C)O)C(C)C	darkorange	0.13191676
ICO	O=C(O)c1c[nH]c2ccccc12	darkorange	0.13176881
DFV	CCCCC(=O)OCC(=O)[C@H]1[C@@H](C[C@@H]2[C@@]1(C[C@@H]([C@]3([C@H]2C[C@@H](C4=CC(=O)C=C[C@@]43C)F)F)O)C)C	darkorange	0.117662159
Izoforon	CC1=CC(=O)CC(C1)(C)C	darkorange	0.159556349
OCT	C[C@@H]([C@H]1CC[C@@H]\2[C@@]1(CCC/C2=C\C=C/3\C[C@H](C[C@@H](C3=C)O)O)C)OCCC(C)(C)O	darkorange	0.111430464
ISOHEPTANE	CCCCC(C)C	darkorange	0.167199114
Heptan	CCCCCCC	darkorange	0.182441507
21987_FLUKA	C[C@H]1C=C[C@@H]2[C@H](C1)C2(C)C	darkorange	0.126515971
EB	CCc1ccccc1	darkorange	0.165613882
butylated hydroxytoluene	[2H]C1=C(C(=C(C(=C1C(C([2H])([2H])[2H])(C([2H])([2H])[2H])C([2H])([2H])[2H])O[2H])C(C([2H])([2H])[2H])(C([2H])([2H])[2H])C([	darkorange	0.131838847

	2H])([2H])[2H])[2H])C([2H])([2H])[2H]		
BuOH	CCCCO	darkorange	0.189682388
sitosterol	CC[C@H](CC[C@@H](C)[C@H]1CC[C@@H]2[C@@]1(CC[C@H]3[C@H]2CC=C4[C@@]3(CC[C@@H](C4)O)C)C)C(C)C	darkorange	0.125221766
2-Caren-10-al	CC1(C2C1C=C(CC2)C=O)C	darkorange	0.111970159
Methylheptane	CCCCCC(C)C	darkorange	0.172315115
Castanin	C[C@H]1[C@H]2[C@](CCC1=O)([C@H]([C@H](C3=C(C(=O)O[C@]23O)C)O)OC(=O)C)C	darkorange	0.108593115
beta-Terpinene	CC(C)C1=CCC(=C)CC1	darkorange	0.18241959
anethole	C/C=C/C1=CC=C(C=C1)OC	darkorange	0.127105293
(E)-1-butoxyhex-2-ene	CCCCOC/C=C/CCC	darkorange	0.124033668
glucuronic acid	[C@@H]1([C@@H]([C@H](OC([C@@H]1O)O)C(=O)O)O)O	darkorange	0.185911216
Octadiene	CCCC/C=C/C=C	darkorange	0.146917164
WLN: 4OVR	CCCCOC(=O)C1=CC=CC=C1	darkorange	0.138661005
Karenzu DK2	C1=CC=C(C=C1)C(=O)CC(=O)C2=CC=CC=C2	darkorange	0.141483826
(1S,2S)-1,2-dimethylcyclopentane	C[C@H]1CCC[C@@H]1C	darkorange	0.159564261
2,2-DIMETHYLPENTANE	CCCC(C)(C)C	darkorange	0.200316125
2,3-dimethylhexane	CCCC(C)C(C)C	darkorange	0.194485005
(3S)-2,3-dimethylpentane	CC[C@H](C)C(C)C	darkorange	0.192746157
5,6,7,8-Tetrahydro-2,4-dimethylquinoline	CC1=CC(=NC2=C1CCCC2)C	darkorange	0.120459341
(4S)-2,4-dimethylhexane	CC[C@H](C)CC(C)C	darkorange	0.164820293
HEX	C1CC(C(=O)N(C1)O)P(=O)(O)O	darkorange	0.135622378
Sextone B	CC1CCCCC1	darkorange	0.154196234

Methylcyclopentane	CC1CCCC1	darkorange	0.151510089
Docosyl caffeate	CCCCCCCCCCCCCCCCCCCCCCOC(=O)/C=C/C1=CC(=C(C=C1)O)O	darkorange	0.119080858
2-methyl-6-ethyl decane	CCCCC(CC)CCCC(C)C	darkorange	0.147979067
Pentadecanol	CCCCCCCCCCCCCCCO	darkorange	0.17943937
Isohexane	CCCC(C)C	darkorange	0.18380874
uralenneoside	C1[C@H]([C@@H]([C@H]([C@@H](O1)OC(=O)C2=CC(=C(C=C2)O)O)O)O)O	darkorange	0.105127513
(E)-dodec-2-ene	CCCCC.CCCCCC.CCCCCCC.CCCCCCCC.CCCCCCCCC.CCCCCCCCCCCC.CCCCCCCCC/C=C/C.CCCCCCC(C)(C)C.CCCCCCC(F)(F)F.CCCCCC1CCCCC1.CCCCCC1CCCC1.CCCCCC1=CC=CC=C1.CCCCCC(C)C.CCCC/C=C/C.CCCCC(C)CC	darkorange	0.116404506
Cyclobutanol, 1-ethyl-	CCC1(CCC1)O	darkorange	0.17984377
2-Tetradecanone	CCCCCCCCCCCCC(=O)C	darkorange	0.154087032
3,3-Dimethylpentane	CCC(C)(C)CC	darkorange	0.198875736
2-Ethyl-p-xylene	CCC1=C(C=CC(=C1)C)C	darkorange	0.17858198
3-methylheptane	CCCCC(C)CC	darkorange	0.15832641
3-methylhexane	CCCC(C)CC	darkorange	0.172136129
3-Methylpentane	CCC(C)CC	darkorange	0.180786826
3-Ethylpentane	CCC(CC)CC	darkorange	0.196244168
2,6,10-trimethyl-dodecane	CCC(C)CCCC(C)CCCC(C)C	darkorange	0.14867203
5,6,7,8-Tetrahydro-4-methylquinoline	CC1=C2CCCCC2=NC=C1	darkorange	0.126626737
icos-5-enoic acid	CCCCCCCCCCCCCCC=CCCCC(=O)O	darkorange	0.126911161
12-methyltetradecanoate	CCC(C)CCCCCCCCCCC(=O)[O-]	darkorange	0.127554046

gadelaidic acid	CCCCCCCCCC/C=C/CCCCCCCC(=O)O	darkorange	0.127739802
Mipax	COC(=O)C1=CC=CC=C1C(=O)OC	darkorange	0.142481027
DIBP	CC(C)COC(=O)C1=CC=CC=C1C(=O)OCC(C)C	darkorange	0.133024804
PENTYLFURAN	CCCCCC1=CC=CO1	darkorange	0.172010563
()-Menthol	C[C@@H]1CC[C@H]([C@@H](C1)O)C(C)C	darkorange	0.146579818
DBP	CCCCOC(=O)c1ccccc1C(=O)OCCCC	darkorange	0.138464758
2-heptanone	CCCCCC(=O)C	darkorange	0.149829471
WLN: VH6	CCCCCCC=O	darkorange	0.162569312
Inermine	C1[C@@H]2[C@H](C3=C(O1)C=C(C=C3)O)OC4=CC5=C(C=C24)OCO5	green	0.11794432
Medicarpin	COC1=CC2=C(C=C1)[C@@H]3COC4=C([C@@H]3O2)C=CC(=C4)O	green	0.115257005
2',7-Dihydroxy-4'-methoxyisoflavan-7-O-β-d-glucopyranoside	CO[C@H]1c2ccc(O[C@@H]3O[C@H](CO)[C@@H](O)[C@H](O)[C@H]3O)cc2O[C@@H](O)[C@@H]1c1ccccc1	green	0.086007388
glyasperin B	CC(=CCC1=C(C=C2C(=C1O)C(=O)C(CO2)C3=C(C=C(C=C3)O)O)OC)C	green	0.114582424
glyasperin F	CC1(C=CC2=C(C=CC(=C2O1)C3COC4=CC(=CC(=C4C3=O)O)O)O)C	green	0.121682439
Glyasperin C	CC(=CCC1=C(C2=C(C=C1O)OC[C@H](C2)C3=C(C=C(C=C3)O)O)OC)C	green	0.134921334
glyasperins D	CC(=CCC1=C(C=C2C(=C1OC)C[C@@H](CO2)C3=C(C=C(C=C3)O)O)OC)C	green	0.14212137
glyasperins Z	COc1ccc([C@H]2COc3cc(O)ccc3C2)c(O)c1CC=C(C)C	green	0.162283584
kanzonols X	CC(=CCC1=C(C=CC(=C1O)[C@H]2CC3=C(C(=C(C=C3)O)CC=C(C)C)OC2)O)C	green	0.168709228
licoagropin	CC(C)=CCc1c(C)ccc2c1OC[C@H]1c3ccc(C)cc3O[C@@H]21	green	0.152088975

Phaseolinisoflavan	CC1(C=CC2=C(O1)C=CC(=C2O)[C@H]3CC4=C(C=C(C=C4)O)OC3)C	green	0.161029694
Licoricidin	CC(=CCC1=C(C=CC(=C1O)[C@H]2CC3=C(C=C(C(=C3OC)CC=C(C)C)O)OC2)O)C	green	0.14004529
licoisoflavanone	CC1(C=CC2=C(O1)C=CC(=C2O)C3COC4=CC(=CC(=C4C3=O)O)O)C	green	0.128202427
shinpterocarpin	CC1(C=CC2=C(O1)C=CC3=C2OC[C@@H]4[C@H]3OC5=C4C=CC(=C5)O)C	green	0.144667191
Hispaglabridin B	CC1(C=CC2=C(O1)C=CC(=C2O)[C@H]3CC4=C(C5=C(C=C4)OC(C=C5)(C)C)OC3)C	green	0.173400128
Glabridin	CC1(C=CC2=C(O1)C=CC3=C2OC[C@H](C3)C4=C(C=C(C=C4)O)O)C	green	0.168251724
(-)-Medicocarpin	COC1=CC2=C(C=C1)[C@@H]3COC4=C([C@@H]3O2)C=CC(=C4)O[C@H]5[C@@H]([C@H]([C@@H]([C@H](O5)CO)O)O)O)O	green	0.09462257
Hispaglabridin A	CC(=CCC1=C(C=CC(=C1O)[C@H]2CC3=C(C4=C(C=C3)OC(C=C4)(C)C)OC2)O)C	green	0.181463464
1-Methoxyphaseollidin	CC(=CCC1=C(C=CC2=C1O[C@@H]3[C@H]2COC4=C3C(=CC(=C4)O)OC)O)C	green	0.150115742
3'(γ,γ-dimethylallyl)-kievitone	CC(C)=CCc1c(O)ccc([C@H]2COc3c(CC=C(C)C)c(O)cc(O)c3C2=O)c1O	green	0.13977841
3'-Hydroxy-4'-O-Methylglabridin	CC1(C=CC2=C(O1)C=CC3=C2OC[C@H](C3)C4=C(C(=C(C=C4)OC)O)O)C	green	0.176004766
3'-Methoxyglabridin	CC1(C=CC2=C(O1)C=CC3=C2OCC(C3)C4=C(C(=C(C=C4)O)OC)O)C	green	0.180980111
1-Methoxyficifolinol	CC(=CCC1=CC2=C(C=C1O)O[C@@H]3[C@H]2COC4=C3C(=C(C(=C4)O)CC=C(C)C)OC)C	green	0.125189952
2-[(3R)-8,8-dimethyl-3,4-dihydro-2H-pyrano[6,5-f]chromen-	COc1ccc([C@@H]2COc3c(ccc4c3C=CC(C)(C)O4)C2)c(O)c1	green	0.172713796

3-yl]-5-methoxyphenol			
Licoriisoflavan A	CC(=CCC1=C(C=CC(=C1O)[C@H]2CC3=C(C(=C(C=C3OC2)OC)CC=C(C)C)OC)O)C	green	0.147713866
Kanzonol F	CC(=CCC1=C(C2=C(C=C1O)OC[C@@H]3[C@H]2OC4=C3C=C5C=CC(OC5=C4)(C)C)OC)C	green	0.120374282
Kanzonol H	CC(=CCC1=C(C=CC(=C1O)[C@H]2CC3=C(C=C4C(=C3OC)CCC(O4)(C)C)OC2)O)C	green	0.126827382
Vestitol	COC1=CC(=C(C=C1)C2CC3=C(C=C(C=C3)O)OC2)O	green	0.148792488
Licoagrocarpin	CC(=CCC1=C(C=CC2=C1OC[C@@H]3[C@H]2OC4=C3C=CC(=C4)OC)O)C	green	0.154527467
Glyasperins K	CC(=CCC1=C(C=C2C(=C1O)C(=O)[C@H](CO2)C3=C(C=C(C=C3)OC)O)OC)C	green	0.112671039
Glyasperins M	CC1(C=CC2=C(O1)C=CC(=C2O)C3COC4=C(C3=O)C(=CC(=C4)O)OC)C	green	0.130754363
Vicenin-2	C1=CC(=CC=C1C2=CC(=O)C3=C(C(=C(C(=C3O2)[C@H]4[C@@H]([C@H]([C@@H]([C@H](O4)CO)O)O)O)O)[C@H]5[C@@H]([C@H]([C@@H]([C@H](O5)CO)O)O)O)O)O	pink	1.047954256
vitexin	C1=CC(=CC=C1C2=CC(=O)C3=C(O2)C(=C(C=C3O)O)[C@H]4[C@@H]([C@H]([C@@H]([C@H](O4)CO)O)O)O)O	pink	0.674515579
violanthin	C[C@H]1[C@@H]([C@H]([C@H]([C@@H](O1)C2=C3C(=C(C(=C2O)[C@H]4[C@@H]([C@H]([C@@H]([C@H](O4)CO)O)O)O)O)C(=O)C=C(O3)C5=CC=C(C=C5)O)O)O)O	pink	0.806859156
schaftoside	C1[C@@H]([C@@H]([C@H]([C@@H](O1)C2=C3C(=C(C(=C2O)[C@H]4[C@@H]([C@H]([C@@H]([C@H](O4)CO)O)O)O)O)C(=O)C=C(O3)C5=CC=C(C=C5)O)O)O)O	pink	0.78202863

Isoviolanthin	C[C@H]1[C@@H]([C@H]([C@H]([C@@H](O1)C2=C(C(=C3C(=C2O)C(=O)C=C(O3)C4=CC=C(C=C4)O)[C@H]5[C@@H]([C@H]([C@@H]([C@H](O5)CO)O)O)O)O)O)O)O	pink	0.823532743
Isoschaftoside	C1[C@@H]([C@@H]([C@H]([C@@H](O1)C2=C(C(=C3C(=C2O)C(=O)C=C(O3)C4=CC=C(C=C4)O)[C@H]5[C@@H]([C@H]([C@@H]([C@H](O5)CO)O)O)O)O)O)O)O	pink	0.787221161
Morusin	CC(=CCC1=C(OC2=C(C1=O)C(=CC3=C2C=CC(O3)(C)C)O)C4=C(C=C(C=C4)O)O)C	red	0.092492024
Glycyrol	CC(=CCC1=C(C2=C(C=C1O)OC(=O)C3=C2OC4=C3C=CC(=C4)O)OC)C	red	0.116557341
Jaranol	COC1=CC(=C2C(=C1)OC(=C(C2=O)OC)C3=CC=C(C=C3)O)O	red	0.102628737
Neouralenol	CC(=CCC1=C(C=CC(=C1O)O)C2=C(C(=O)C3=CC(=C(C=C3O2)O)O)O)C	red	0.132493586
isorhamnetin	COC1=C(C=CC(=C1)C2=C(C(=O)C3=C(C=C(C=C3O2)O)O)O)O	red	0.096726622
Lupiwighteone	CC(=CCC1=C2C(=C(C=C1O)O)C(=O)C(=CO2)C3=CC=C(C=C3)O)C	red	0.112937352
formononetin	COC1=CC=C(C=C1)C2=COC3=C(C2=O)C=CC(=C3)O	red	0.105615408
Scopoletol	COC1=C(C=C2C(=C1)C=CC(=O)O2)O	red	0.097282987
Calycosin	COC1=C(C=C(C=C1)C2=COC3=C(C2=O)C=CC(=C3)O)O	red	0.109979252
kaempferol	C1=CC(=CC=C1C2=C(C(=O)C3=C(C=C(C=C3O2)O)O)O)O	red	0.104201502
8-Prenylwighteone	CC(=CCC1=C(C(=C2C(=C1O)C(=O)C(=CO2)C3=CC=C(C=C3)O)CC=C(C)C)O)C	red	0.112820541
glyasperin E	COc1cc2oc(Oc3ccc(O)cc3O)c(-c3ccccc3)c(=O)c2cc1CC=C(C)C	red	0.083886213
Isotrifoliol	COC1=CC(=CC2=C1C3=C(C4=C(O3)C=C(C=C4)O)C(=O)O2)O	red	0.09888694

kanzonols K	CC(=CCC1=C(C=CC(=C1O)C2=COC3=CC(=C(C(=C3C2=O)O)CC=C(C)C)OC)O)C	red	0.137655058
kanzonols L	CC(=CCC1=C(C(=C2C(=C1O)C(=O)C(=CO2)C3=C4C(=C(C=C3)O)C=CC(O4)(C)C)CC=C(C)C)O)C	red	0.108205898
kanzonols T	CC1(C=CC2=C(O1)C=CC(=C2O)C3=COC4=C(C3=O)C(=C(C(=C4)O)CCC(C)(C)O)O)C	red	0.097718778
kanzonols W	CC1(C=CC2=C(O1)C=CC3=C2OC(=O)C(=C3)C4=C(C=C(C=C4)O)O)C	red	0.081927711
(2S)-6-(2,4-dihydroxyphenyl)-2-(2-hydroxypropan-2-yl)-4-methoxy-2,3-dihydrofuro[3,2-g]chromen-7-one	CC(C)([C@@H]1CC2=C(O1)C=C3C(=C2OC)C=C(C(=O)O3)C4=C(C=C(C=C4)O)O)O	red	0.088443124
Semilicoisoflavone B	CC1(C=CC2=C(O1)C(=CC(=C2)C3=COC4=CC(=CC(=C4C3=O)O)O)O)C	red	0.112118155
Glepidotin A	CC(=CCC1=C2C(=C(C=C1O)O)C(=O)C(=C(O2)C3=CC=CC=C3)O)C	red	0.121051245
3-(2-hydroxy-4-methoxyphenyl)-2H-chromen-7-ol	COC1=CC(=C(C=C1)C2=CC3=C(C=C(C=C3)O)OC2)O	red	0.07286876
8-(6-hydroxy-2-benzofuranyl)-2,2-dimethyl-5-chromenol	CC1(C)C=Cc2c(O)ccc(-c3cc4ccc(O)cc4o3)c2O1	red	0.075776073
Liconeolignan	CC(=CCC1=C(C=CC(=C1)C2=C(C3=CC(=C(C=C3O2)OC)OC)O)O)C	red	0.103324054
3-(2,4-dihydroxyphenyl)-8-(1,1-dimethylprop-2-enyl)-7-hydroxy-5-methoxy-coumarin	C=CC(C)(C)c1c(O)cc(OC)c2cc(-c3ccc(O)cc3O)c(=O)oc12	red	0.103572564
Licoflavone	CC(=CCC1=CC2=C(C=C1O)OC(=CC2=O)C3=CC=C(C=C3)O)C	red	0.088572731
7-hydroxy-2-[4-hydroxy-3-(3-methylbut-2-enyl)phenyl]-6-(3-	CC(C)=CCc1cc(-c2cc(=O)c3cc(CC=C(C)C)c(O)cc3o2)ccc1O	red	0.091681419

methylbut-2-enyl)chromone			
Licoflavonol	CC(=CCC1=C(C2=C(C=C1O)OC(=C(C2=O)O)C3=CC=C(C=C3)O)O)C	red	0.123649578
Yinyanghuo D	CC(=CCC1=C(C=CC(=C1)C2=CC(=O)C3=C(C=C(C=C3O2)O)O)O)C	red	0.101835871
Licoricone	CC(=CCC1=C(C=C(C(=C1OC)C2=COC3=C(C2=O)C=CC(=C3)O)O)OC)C	red	0.136174598
Gancaonin A	CC(=CCC1=C(C2=C(C=C1O)OC=C(C2=O)C3=CC=C(C=C3)OC)O)C	red	0.130976186
Gancaonin B	CC(=CCC1=C(C2=C(C=C1O)OC=C(C2=O)C3=CC(=C(C=C3)OC)O)O)C	red	0.134322854
Gancaonin C	C/C(=C\CC1=C2C(=C(C=C1O)O)C(=O)C(=CO2)C3=CC=C(C=C3)O)/CO	red	0.105624485
Prunetin	COC1=CC(=C2C(=C1)OC=C(C2=O)C3=CC=C(C=C3)O)O	red	0.114507158
Gancaonin D	C/C(=C\CC1=C2C(=C(C=C1O)O)C(=O)C(=CO2)C3=CC(=C(C=C3)OC)O)/CO	red	0.103236562
3-(3,4-dihydroxyphenyl)-5,7-dihydroxy-8-(3-methylbut-2-enyl)chromone	CC(C)=CCc1c(O)cc(O)c2c(=O)c(-c3ccc(O)c(O)c3)coc12	red	0.11449599
5,7-dihydroxy-3-(4-methoxyphenyl)-8-(3-methylbut-2-enyl)chromone	COc1ccc(-c2coc3c(CC=C(C)C)c(O)cc(O)c3c2=O)cc1	red	0.120476598
5,7-dihydroxy-3-(2-hydroxy-4-methoxy-phenyl)-6-(3-methylbut-2-enyl)chromone	COc1ccc(-c2coc3cc(O)c(CC=C(C)C)c(O)c3c2=O)c(O)c1	red	0.131132561
2-(3,4-dihydroxyphenyl)-5,7-dihydroxy-6-(3-methylbut-2-enyl)chromone	CC(C)=CCc1c(O)cc2oc(-c3ccc(O)c(O)c3)cc(=O)c2c1O	red	0.105866385

Gancaonin P	CC(=CCC1=C(C2=C(C=C1O)OC(=C(C2=O)O)C3=CC(=C(C=C3)O)O)O)C	red	0.124225303
Gancaonin Q	CC(=CCC1=C(C=CC(=C1)C2=CC(=O)C3=C(O2)C=C(C(=C3O)CC=C(C)C)O)O)C	red	0.103695699
3-[4,6-dihydroxy-2-methoxy-3-(3-methylbut-2-enyl)phenyl]-7-hydroxy-chromone	COc1c(CC=C(C)C)c(O)cc(O)c1-c1coc2cc(O)ccc2c1=O	red	0.136811442
Glycycoumarin	CC(=CCC1=C(C2=C(C=C1O)OC(=O)C(=C2)C3=C(C=C(C=C3)O)O)OC)C	red	0.124516309
Glycyrin	CC(=CCC1=C(C=C2C(=C1OC)C=C(C(=O)O2)C3=C(C=C(C=C3)O)O)OC)C	red	0.123501712
Licocoumarone	CC(=CCC1=C(C2=C(C=C1O)OC(=C2)C3=C(C=C(C=C3)O)O)OC)C	red	0.132998553
Licoisoflavone	CC(=CCC1=C(C=CC(=C1O)C2=COC3=CC(=CC(=C3C2=O)O)O)O)C	red	0.13448577
Licoisoflavone B	CC1(C=CC2=C(O1)C=CC(=C2O)C3=COC4=CC(=CC(=C4C3=O)O)O)C	red	0.097201276
licopyranocoumarin	CC1(CCC2=C(O1)C=C3C(=C2OC)C=C(C(=O)O3)C4=C(C=C(C=C4)O)O)CO	red	0.080516206
Glyzaglabrin	C1OC2=C(O1)C(=C(C=C2)C3=COC4=C(C3=O)C=CC(=C4)O)O	red	0.09679194
Glabrene	CC1(C=CC2=C(C=CC(=C2O1)C3=CC4=C(C=C(C=C4)O)OC3)O)C	red	0.07349858
Glabrone	CC1(C=CC2=C(O1)C=CC(=C2O)C3=COC4=C(C3=O)C=CC(=C4)O)C	red	0.095031063
1,3-dihydroxy-9-methoxy-6-benzofurano[3,2-c]chromenone	COc1ccc2c(c1)oc1c3c(O)cc(O)cc3oc(=O)c21	red	0.097635026
1,3-dihydroxy-8,9-dimethoxy-6-benzofurano[3,2-c]chromenone	COc1cc2oc3c4c(O)cc(O)cc4oc(=O)c3c2cc1OC	red	0.09431335

Eurycarpin A	CC(=CCC1=C(C=CC(=C1O)C2=COC3=C(C2=O)C=CC(=C3)O)O)C	red	0.134634289
Uralenol	CC(=CCC1=C(C(=CC(=C1)C2=C(C(=O)C3=C(C=C(C=C3O2)O)O)O)O)O)C	red	0.113359375
Uralenol-3-methylether	CC(=CCC1=C(C(=CC(=C1)C2=C(C(=O)C3=C(C=C(C=C3O2)O)O)OC)O)O)C	red	0.118023838
Uralene	CC(=CCC1=CC(=C(C=C1C2=C(C(=O)C3=C(O2)C=CC(=C3O)O)OC)O)O)C	red	0.127864085
Isoglycyrol	CC1(CCC2=C(O1)C=C3C(=C2OC)C4=C(C5=C(O4)C=C(C=C5)O)C(=O)O3)C	red	0.083975663
Isolicoflavonol	CC(=CCC1=C(C=CC(=C1)C2=C(C(=O)C3=C(C=C(C=C3O2)O)O)O)O)C	red	0.111879351
isoglycycoumarin	CC1(CCC2=C(O1)C=C3C(=C2OC)C=C(C(=O)O3)C4=C(C=C(C=C4)O)O)C	red	0.082898814
HMO	COc1ccc2c(=O)c(-c3ccc(O)cc3)coc2c1	red	0.1043217
Quercetin der.	COc1cc(-c2oc3cc(O)cc(O)c3c(=O)c2OC)ccc1O	red	0.096192492
Daidzein dimethyl ether	COC1=CC=C(C=C1)C2=COC3=C(C2=O)C=CC(=C3)OC	red	0.098737435
Inflacoumarin A	CC(C)=CCc1cc2c(-c3ccc(O)cc3)cc(=O)oc2cc1O	red	0.08630536
7,2',4'-trihydroxy－5-methoxy-3－arylcoumarin	COC1=CC(=CC2=C1C=C(C(=O)O2)C3=C(C=C(C=C3)O)O)O	red	0.103651073
Artonin E	CC(=CCC1=C(OC2=C(C1=O)C(=CC3=C2C=CC(O3)(C)C)O)C4=CC(=C(C=C4O)O)O)C	red	0.092554963
Gancaonin G	CC(=CCC1=C(C=C2C(=C1O)C(=O)C(=CO2)C3=CC=C(C=C3)O)OC)C	red	0.138815043
Gancaonin H	CC(=CCC1=C(C2=C(C=C1O)OC=C(C2=O)C3=CC4=C(C(=C3)O)OC(C=C4)(C)C)O)C	red	0.115908683
Gancaonin I	CC(=CCC1=C(C=C2C(=C1OC)C=C(O2)C3=C(C=C(C=C3)O)O)OC)C	red	0.13368363

Glyasperin A	CC(=CCC1=C(C=CC(=C1)C2=C(C(=O)C3=C(O2)C=C(C(=C3O)CC=C(C)C)O)O)O)C	red	0.11852305
Glycyrrhiza flavonol A	CC1(C(CC2=C(O1)C=CC(=C2)C3=C(C(=O)C4=C(C=C(C=C4O3)O)O)O)O)C	red	0.091812581
Kanzonol E	CC(=CCC1=CC2=C(C=C1O)OC(=CC2=O)C3=CC4=C(C=C3)OC(C=C4)(C)C)C	red	0.083846246
Licoagroisoflavone	CC(=C)[C@H]1CC2=C(O1)C=C3C(=C2O)C(=O)C(=CO3)C4=CC=C(C=C4)O	red	0.117103422
Odoratin	COC1=C(C=C(C=C1)C2=COC3=CC(=C(C=C3C2=O)OC)O)O	red	0.107777418
Phaseol	CC(=CCC1=C(C=CC2=C1OC(=O)C3=C2OC4=C3C=CC(=C4)O)O)C	red	0.117760173
dehydroglyasperins C	CC(C)=CCc1c(O)cc2c(c1O)C=C(c1ccc(O)cc1O)CO2	red	0.076871073
quercetin	C1=CC(=C(C=C1C2=C(C(=O)C3=C(C=C(C=C3O2)O)O)O)O)O	red	0.100361283
isoliquiritigenin	C1=CC(=CC=C1/C=C/C(=O)C2=C(C=C(C=C2)O)O)O	blue	0.332180491
(E)-1-(2,4-dihydroxyphenyl)-3-(2,2-dimethylchromen-6-yl)prop-2-en-1-one	CC1(C=CC2=C(O1)C=CC(=C2)/C=C/C(=O)C3=C(C=C(C=C3)O)O)C	blue	0.314256507
(2R)-1-[2,4-dihydroxy-5-(3-methylbut-2-enyl)phenyl]-2-hydroxy-3-[4-hydroxy-3-(3-methylbut-2-enyl)phenyl]propan-1-one	CC(=CCC1=CC(=C(C=C1O)O)C(=O)[C@@H](CC2=CC(=C(C=C2)O)CC=C(C)C)O)C	blue	0.21656859
(E)-1-(2,4-dihydroxyphenyl)-3-[4-hydroxy-3-(3-methylbut-2-enyl)phenyl]prop-2-en-1-one	CC(=CCC1=C(C=CC(=C1)/C=C/C(=O)C2=C(C=C(C=C2)O)O)O)C	blue	0.292824335
glyinflanin A	CC(=CCC1=CC(=C(C=C1O)O)C(=O)CC(=O)C2=CC(=C(C=C2)O)CC=C(C)C)C	blue	0.207539533

(E)-1-[2,4-dihydroxy-3-(3-methylbut-2-enyl)phenyl]-3-[4-hydroxy-3-(3-methylbut-2-enyl)phenyl]prop-2-en-1-one	CC(=CCC1=C(C=CC(=C1)/C=C/C(=O)C2=C(C(=C(C=C2)O)CC=C(C)C)O)O)C	blue	0.249408564
(E)-1-[2,4-dihydroxy-3-(3-methylbut-2-enyl)phenyl]-3-(2,4-dihydroxyphenyl)prop-2-en-1-one	CC(=CCC1=C(C=CC(=C1O)C(=O)/C=C/C2=C(C=C(C=C2)O)O)O)C	blue	0.227214696
(E)-3-[3,4-dihydroxy-5-(3-methylbut-2-enyl)phenyl]-1-(2,4-dihydroxyphenyl)prop-2-en-1-one	CC(=CCC1=C(C(=CC(=C1)/C=C/C(=O)C2=C(C=C(C=C2)O)O)O)O)C	blue	0.31563274
neoisoliquiritin	C1=CC(=CC=C1/C=C/C(=O)C2=C(C=C(C=C2)O[C@H]3[C@@H]([C@H]([C@@H]([C@H](O3)CO)O)O)O)O)O	blue	0.234715232
Isoliquiritin	C1=CC(=CC=C1/C=C/C(=O)C2=C(C=C(C=C2)O)O)O[C@H]3[C@@H]([C@H]([C@@H]([C@H](O3)CO)O)O)O	blue	0.240032913
licuraside	C1[C@@]([C@H]([C@@H](O1)O[C@@H]2[C@H]([C@@H]([C@H](O[C@H]2OC3=CC(=C(C=C3)C(=O)/C=C/C4=CC=C(C=C4)O)O)CO)O)O)O)(CO)O	blue	0.194760852
(Z)-1-(2,4-dihydroxyphenyl)-3-phenylprop-2-en-1-one	C1=CC=C(C=C1)/C=C\C(=O)C2=C(C=C(C=C2)O)O	blue	0.322643265
4,2',4',alpha-Tetrahydroxydihydrochalcone	C1=CC(=CC=C1C[C@H](C(=O)C2=C(C=C(C=C2)O)O)O)O	blue	0.250530047
1-(5-hydroxy-2,2-dimethylchromen-6-yl)-3-(4-hydroxyphenyl)prop-2-en-1-one	CC1(C)C=Cc2c(ccc(C(=O)C=Cc3ccc(O)cc3)c2O)O1	blue	0.290028962
Corylifolinin	CC(=CCC1=C(C=CC(=C1O)C(=O)/C=C/C2=CC=C(C=C2)O)O)C	blue	0.254116705

Licorice glycoside A	COC1=C(C=CC(=C1)/C=C/C(=O)OC[C@]2(CO[C@H]([C@@H]2O)O[C@@H]3[C@H]([C@@H]([C@H](O[C@H]3OC4=CC=C(C=C4)/C=C/C(=O)C5=C(C=C(C=C5)O)O)CO)O)O)O)O	blue	0.171838552
Mairin	CC(=C)[C@@H]1CC[C@]2([C@H]1[C@H]3CC[C@@H]4[C@]5(CC[C@@H](C([C@@H]5CC[C@]4([C@@]3(CC2)C)C)(C)C)O)C)C(=O)O	gold	0.134782088
oleanolic acid	C[C@]12CC[C@@H](C([C@@H]1CC[C@@]3([C@@H]2CC=C4[C@]3(CC[C@@]5([C@H]4CC(CC5)(C)C)C(=O)O)C)C)(C)C)O	gold	0.171398718
18beta-glycyrrhetinic acid	C[C@]12CC[C@](C[C@H]1C3=CC(=O)[C@@H]4[C@]5(CC[C@@H](C([C@@H]5CC[C@]4([C@@]3(CC2)C)C)(C)C)O)C)(C)C(=O)O	gold	0.243574653
apioglycyrrhizin	CC1(C2CCC3(C(C2(CCC1OC4C(C(C(C(O4)C(=O)O)O)O)OC5C(C(CO5)(CO)O)O)C)C(=O)C=C6C3(CCC7(C6CC(CC7)(C)C(=O)O)C)C)C)C	gold	0.157594014
apioglycyrrhizin_qt	CC1(C)[C@@H](O)CC[C@]2(C)[C@@H]3C(=O)C=C4[C@@H]5C[C@@](C)(C(=O)O)CC[C@@]5(C)CC[C@]4(C)[C@@]3(C)CC[C@@H]12	gold	0.243574653
liquoric acid	C[C@]12CC[C@@H](C(C1CC[C@@]3(C2C(=O)C=C4[C@]3(C[C@@H]5[C@@]6([C@H]4C[C@@]([C@@H](C6)O5)(C)C(=O)O)C)C)C)(C)C)O	gold	0.194487639
Glycyram	C[C@]12CC[C@](C[C@H]1C3=CC(=O)[C@@H]4[C@]5(CC[C@@H](C([C@@H]5CC[C@]4([C@@]3(CC2)C)C)(C)C)O[C@@H]6[C@@H]([C@H]([C@@H]([C@H](O6)C(=O)O)O)O)O[C@H]7[C@@H]([C@H]([C@@H]([C@H](O7)C(=O)O)O)O)O)C)(C)C(=O)O.N	gold	0.185603356
licorice-saponin C2	C[C@]12CC[C@](CC1=C3C=C[C@@H]4[C@]5(CC[C@@H](C([C	gold	0.154852039

	@@H]5CC[C@]4([C@@]3(CC2)C)C)(C)C)O[C@H]6[C@@H]([C@H]([C@@H]([C@H](O6)C(=O)O)O)O)O[C@H]7[C@@H]([C@H]([C@@H]([C@H](O7)C(=O)O)O)O)O)C)(C)C(=O)O		
licorice-saponin C2_qt	CC1(C)[C@@H](O)CC[C@@]2(C)[C@H]1CC[C@]1(C)[C@@H]2C=CC2=C3C[C@@](C)(C(=O)O)CC[C@]3(C)CC[C@]21C	gold	0.145429788
licorice-saponin F3	CC1(C)[C@H](O[C@H]2O[C@H](C(=O)O)[C@@H](O)[C@@H](O)[C@H]2O[C@@H]2O[C@H](C(=O)O)[C@@H](O)[C@@H](O)[C@H]2O[C@@H]2O[C@H](C(=O)O)[C@@H](O)[C@@H](O)[C@H]2O)CC[C@@]2(C)[C@H]1CC[C@]1(C)[C@@H]2CC=C2[C@H]3C[C@@]4(C)C[C@@H](OC4=O)[C@]3(C)CC[C@@]21C	gold	0.209285563
licorice-saponin F3_qt	CC1(C)[C@@H](O)CC[C@]2(C)[C@@H]3CC=C4[C@@H]5C[C@@]6(C)C[C@H](OC6=O)[C@@]5(C)CC[C@@]4(C)[C@@]3(C)CC[C@@H]12	gold	0.202011971
licorice-saponin G2	C[C@]12CC[C@](C[C@H]1C3=CC(=O)[C@@H]4[C@]5(CC[C@@H]([C@]([C@@H]5CC[C@]4([C@@]3(CC2)C)C)(C)CO)O[C@H]6[C@@H]([C@H]([C@@H]([C@H](O6)C(=O)O)O)O)O[C@H]7[C@@H]([C@H]([C@@H]([C@H](O7)C(=O)O)O)O)O)C)(C)C(=O)O	gold	0.168328881
licorice-saponin G2_qt	C[C@@]12CC[C@]3(C)C(=CC(=O)[C@H]4[C@@]5(C)CC[C@H](O)[C@@](C)(CO)[C@@H]5CC[C@@]43C)[C@@H]1C[C@](C)(C(=O)O)CC2	gold	0.217532304
licorice-saponin H2	C[C@]12CC[C@@](C[C@H]1C3=CC(=O)[C@@H]4[C@]5(CC[C@@H](C([C@@H]5CC[C@]4([C@@]3(CC2)C)C)(C)C)O[C@H]6[C@@H]([C@H]([C@@H]([C@H](O6)C(=O)O)O)O)O[C@H]7[C@	gold	0.206498532

licorice-saponin H2_qt	@H]([C@H]([C@@H]([C@H](O7)C(=O)O)O)O)O)C)(C)C(=O)O	gold	0.243574653
licorice-saponin J2	CC1(C)[C@@H](O)CC[C@]2(C)[C@@H]3C(=O)C=C4[C@@H]5C[C@@](C)(C(=O)O)CC[C@]5(C)CC[C@@]4(C)[C@@]3(C)CC[C@@H]12	gold	0.171943902
licorice-saponin J2_qt	C[C@]12CC[C@](C[C@H]1C3=CC[C@@H]4[C@]5(CC[C@@H]([C@]([C@@H]5CC[C@]4([C@@]3(CC2)C)C)(C)CO)O[C@H]6[C@@H]([C@H]([C@@H]([C@H](O6)C(=O)O)O)O)O[C@H]7[C@@H]([C@H]([C@@H]([C@H](O7)C(=O)O)O)O)O)C)(C)C(=O)O	gold	0.175620544
licorice-saponin B2	C[C@]1(C(=O)O)CC[C@@]2(C)CC[C@]3(C)C(=CC[C@@H]4[C@]3(C)CC[C@H]3[C@](C)(CO)[C@@H](O)CC[C@]43C)[C@@H]2C1	gold	0.197065812
licorice-saponin K2	C[C@]12CC[C@](C[C@H]1C3=CC[C@@H]4[C@]5(CC[C@@H](C([C@@H]5CC[C@]4([C@@]3(CC2)C)C)(C)C)O[C@H]6[C@@H]([C@H]([C@@H]([C@H](O6)C(=O)O)O)O)O[C@H]7[C@@H]([C@H]([C@@H]([C@H](O7)C(=O)O)O)O)O)C)(C)C(=O)O	gold	0.144442166
licorice-saponin K2_qt	C[C@]12CC[C@](CC1=C3C=C[C@@H]4[C@]5(CC[C@@H]([C@]([C@@H]5CC[C@]4([C@@]3(CC2)C)C)(C)CO)O[C@H]6[C@@H]([C@H]([C@@H]([C@H](O6)C(=O)O)O)O)O[C@H]7[C@@H]([C@H]([C@@H]([C@H](O7)C(=O)O)O)O)O)C)(C)C(=O)O	gold	0.14031408
glycyrrhetol	C[C@@]12CC[C@]3(C)C(=C1C[C@](C)(C(=O)O)CC2)C=C[C@H]1[C@@]2(C)CC[C@H](O)[C@@](C)(CO)[C@@H]2CC[C@@]13C	gold	0.196465332
	C[C@]12CC[C@](C[C@H]1C3=CC(=O)[C@@H]4[C@]5(CC[C@@H](C([C@@H]5CC[C@]4([C@@]3(CC2)C)C)(C)C)O)C)(C)CO		

3,22-Dihydroxy-11-oxo-delta(12)-oleanene-27-alpha-methoxycarbonyl-29-oic acid	COC(=O)[C@@]12CC[C@]3(C)[C@@H](C[C@]4(C)C[C@H]3OC4=O)C1=CC(=O)[C@@H]1[C@@]3(C)CC[C@H](O)C(C)(C)[C@@H]3CC[C@]12C	gold	0.198244355
glabrolide	C[C@]12CC[C@@H](C([C@@H]1CC[C@@]3([C@@H]2C(=O)C=C4[C@]3(CC[C@@]5([C@H]4C[C@@]6(C[C@H]5OC6=O)C)C)C)C)(C)C)O	gold	0.224036519
glycyrrhizin	C[C@]12CC[C@](C[C@H]1C3=CC(=O)[C@@H]4[C@]5(CC[C@@H](C([C@@H]5CC[C@]4([C@@]3(CC2)C)C)(C)C)O[C@@H]6[C@@H]([C@H]([C@@H]([C@H](O6)C(=O)O)O)O)O[C@H]7[C@@H]([C@H]([C@@H]([C@H](O7)C(=O)O)O)O)O)C)(C)C(=O)O	gold	0.206498532
uralsaponin B	CC1(C2CCC3(C(C2(CCC1OC4C(C(C(C(O4)C(=O)O)O)OC5C(C(C(C(O5)C(=O)O)O)O)O)O)C)C(=O)C=C6C3(CCC7(C6CC(CC7)(C)C(=O)O)C)C)C)C	gold	0.215668626
isoglabrolide	CC1(C2CCC3(C(C2(CCC1O)C)C(=O)C=C4C3(CCC5(C46CC(CC5)(C(=O)O6)C)C)C)C)C	gold	0.151339956
22β-acetylglabric acid	CC(=O)O[C@@H]1C[C@](C)(C(=O)O)C[C@@H]2C3=CC(=O)[C@@H]4[C@]5(C)CC[C@@H](O)C(C)(C)[C@H]5CC[C@@]4(C)[C@]3(C)CC[C@@]12C	gold	0.254544815
24-Hydroxy-11-deoxyglycyrrhetic acid	C[C@]1(C(=O)O)CC[C@]2(C)CC[C@]3(C)C(=CC[C@@H]4[C@@]5(C)CC[C@H](O)[C@](C)(O)[C@@H]5CC[C@]43C)[C@@H]2C1	gold	0.198451915
24-Hydroxyglycyrrhetic acid	CC12CCC(CC1C3=CC(=O)C4C5(CCC(C(C5CCC4(C3(CC2)C)C)(C)CO)O)C)(C)C(=O)O	gold	0.217532304
3β-formylglabrolide	CC1(C)[C@H]2CC[C@]3(C)[C@H](C(=O)C=C4[C@H]5C[C@@]6(C)C[C@H](OC6=O)[C@]5(C)CC[C@]43C)[C@]2(C)CC[C@H]1OC=O	gold	0.225335402

11-deoxyglycyrrhetic acid	CC1(C)[C@@H](O)CC[C@@]2(C)[C@H]1CC[C@]1(C)[C@@H]2CC=C2[C@@H]3C[C@@](C)(C(=O)O)CC[C@]3(C)CC[C@]21C	gold	0.198722445
Araboglycyrrhizin	CC1(C2CCC3(C(C2(CCC1OC4C(C(C(C(O4)C(=O)O)O)O)OC5C(C(C(CO5)O)O)O)C)C(=O)C=C6C3(CCC7(C6CC(CC7)(C)C(=O)O)C)C)C)C	gold	0.182825578
Araboglycyrrhizin_qt	CC1(C)[C@@H](O)CC[C@@]2(C)[C@@H]1CC[C@@]1(C)[C@H]2C(=O)C=C2[C@@H]3C[C@@](C)(C(=O)O)CC[C@]3(C)CC[C@]21C	gold	0.243574653
beta-Glycyrrhetinic acid	C[C@]12CC[C@](C[C@@H]1C3=CC(=O)[C@@H]4[C@]5(CC[C@@H](C([C@@H]5CC[C@]4([C@@]3(CC2)C)C)(C)C)O)C)(C)C(=O)O	gold	0.243574653
18α-hydroxyglycyrrhetic acid	CC1(C)[C@@H](O)CC[C@]2(C)[C@@H]3C(=O)C=C4[C@@](C)(CC[C@@]5(C)CC[C@](C)(C(=O)O)C[C@]45O)[C@]3(C)CC[C@@H]12	gold	0.171751894
ursolic acid	C[C@@H]1CC[C@@]2(CC[C@@]3(C(=CC[C@H]4[C@]3(CC[C@@H]5[C@@]4(CC[C@@H](C5(C)C)O)C)C)[C@@H]2[C@H]1C)C)C(=O)O	gold	0.155761004
nicotiflorin	C[C@H]1[C@@H]([C@H]([C@H]([C@@H](O1)OC[C@@H]2[C@H]([C@@H]([C@H]([C@@H](O2)OC3=C(OC4=CC(=CC(=C4C3=O)O)O)C5=CC=C(C=C5)O)O)O)O)O)O)O	gray	0.952909692
Narcissoside	C[C@H]1[C@@H]([C@H]([C@H]([C@@H](O1)OC[C@@H]2[C@H]([C@@H]([C@H]([C@@H](O2)OC3=C(OC4=CC(=CC(=C4C3=O)O)O)C5=CC(=C(C=C5)O)OC)O)O)O)O)O	gray	0.756328448
rutin	C[C@H]1[C@@H]([C@H]([C@H]([C@@H](O1)OC[C@@H]2[C@H]([C@@H]([C@H]([C@@H](O2	gray	1.025207199

	)OC3=C(OC4=CC(=CC(=C4C3=O)O)O)C5=CC(=C(C=C5)O)O)O)O)O)O)O)O		
Hirsutrin	C1=CC(=C(C=C1C2=C(C(=O)C3=C(C=C(C=C3O2)O)O)O[C@H]4[C@@H]([C@H]([C@@H]([C@H](O4)CO)O)O)O)O)O	gray	0.823572944
Astragalin	C1=CC(=CC=C1C2=C(C(=O)C3=C(C=C(C=C3O2)O)O)O[C@H]4[C@@H]([C@H]([C@@H]([C@H](O4)CO)O)O)O)O	gray	0.784666746
Pinocembrin	C1[C@H](OC2=CC(=CC(=C2C1=O)O)O)C3=CC=CC=C3	cyan	0.18960015
naringenin	C1[C@H](OC2=CC(=CC(=C2C1=O)O)O)C3=CC=C(C=C3)O	cyan	0.19072412
3-Hydroxyglabrol	CC(=CCC1=C(C=CC(=C1)[C@@H]2[C@H](C(=O)C3=C(O2)C(=C(C=C3)O)CC=C(C)C)O)O)C	cyan	0.191109655
(2S)-2-[4-hydroxy-3-(3-methylbut-2-enyl)phenyl]-8,8-dimethyl-2,3-dihydropyrano[2,3-f]chromen-4-one	CC(=CCC1=C(C=CC(=C1)[C@@H]2CC(=O)C3=C(O2)C4=C(C=C3)OC(C=C4)(C)C)O)C	cyan	0.200709481
euchrenone	CC(=CCC1=C(C=CC2=C1O[C@@H](CC2=O)C3=C(C=C(C=C3)O)O)O)C	cyan	0.191348891
naringin	C[C@H]1[C@@H]([C@H]([C@H]([C@@H](O1)O[C@@H]2[C@H]([C@@H]([C@H](O[C@H]2OC3=CC(=C4C(=O)C[C@H](OC4=C3)C5=CC=C(C=C5)O)O)CO)O)O)O)O)O	cyan	0.143830606
Glepidotin B	CC(=CCC1=C2C(=C(C=C1O)O)C(=O)[C@@H]([C@H](O2)C3=CC=CC=C3)O)C	cyan	0.129322805
glabrol	CC(=CCC1=C(C=CC(=C1)[C@@H]2CC(=O)C3=C(O2)C(=C(C=C3)O)CC=C(C)C)O)C	cyan	0.207324963
licorice glycoside E	C1[C@H](OC2=C(C1=O)C=CC(=C2)O)C3=CC=C(C=C3)O[C@H]4C([C@H]([C@@H](C(O4)CO)O)O)O)O[C@H]5[C@H](C(CO5)(COC(=O)C6=CNC7=CC=CC=C76)O)O	cyan	0.109147886

(2R)-2-[3,4-dihydroxy-5-(3-methylbut-2-enyl)phenyl]-5,7-dihydroxy-8-(3-methylbut-2-enyl)chroman-4-one	CC(C)=CCc1cc([C@H]2CC(=O)c3c(O)cc(O)c(CC=C(C)C)c3O2)cc(O)c1O	cyan	0.164002995
liquiritin	C1[C@H](OC2=C(C1=O)C=CC(=C2)O)C3=CC=C(C=C3)O[C@H]4[C@@H]([C@H]([C@@H]([C@H](O4)CO)O)O)O	cyan	0.138589905
Glabranin	CC(=CCC1=C2C(=C(C=C1O)O)C(=O)C[C@H](O2)C3=CC=CC=C3)C	cyan	0.144477907
4H-1-Benzopyran-4-one, 2-(4-(beta-D-glucopyranosyloxy)phenyl)-2,3-dihydro-5,7-dihydroxy-, (2S)-	O=C1C[C@@H](c2ccc(O[C@@H]3O[C@H](CO)[C@@H](O)[C@H](O)[C@H]3O)cc2)Oc2cc(O)cc(O)c21	cyan	0.153650456
Sigmoidin-B	CC(=CCC1=C(C(=CC(=C1)[C@@H]2CC(=O)C3=C(C=C(C=C3O2)O)O)O)O)C	cyan	0.170669959
neoliquiritin	C1[C@H](OC2=C(C1=O)C=CC(=C2)O[C@H]3[C@@H]([C@H]([C@@H]([C@H](O3)CO)O)O)O)C4=CC=C(C=C4)O	cyan	0.135597264
(2R)-7-hydroxy-2-(4-hydroxyphenyl)chroman-4-one	O=C1C[C@H](c2ccc(O)cc2)Oc2cc(O)ccc21	cyan	0.196391585
(2S)-7-hydroxy-2-(4-hydroxyphenyl)-8-(3-methylbut-2-enyl)chroman-4-one	CC(C)=CCc1c(O)ccc2c1O[C@H](c1ccc(O)cc1)CC2=O	cyan	0.198522861
Liquiritin apioside	C1[C@H](OC2=C(C1=O)C=CC(=C2)O)C3=CC=C(C=C3)O[C@H]4[C@@H]([C@H]([C@@H]([C@H](O4)CO)O)O)O[C@H]5[C@@H]([C@](CO5)(CO)O)O	cyan	0.11773387
isograbrol	CC(C)=CCc1cc2c(c(CC=C(C)C)c1O)O[C@@H](c1ccc(O)cc1)CC2=O	cyan	0.186553935
6″-O-acetylliquiritin	CC(=O)OC[C@@H]1[C@H]([C@@H]([C@H]([C@@H](O1)OC2=	cyan	0.141590646

	CC=C(C=C2)[C@@H]3CC(=O)C4=C(O3)C=C(C=C4)O)O)O)O		
6-prenylated eriodictyol	CC(C)=CCc1c(O)cc2c(c1O)C(=O)C[C@H](c1ccc(O)c(O)c1)O2	cyan	0.146998208
8-prenylated eriodictyol	CC(C)=CCc1c(O)cc(O)c2c1O[C@@H](c1ccc(O)c(O)c1)CC2=O	cyan	0.153226111
Kanzonol Z	CC(=CCC1=C(C=CC(=C1)[C@@H]2[C@H](C(=O)C3=C(O2)C4=C(C=C3)OC(C=C4)(C)C)O)O)C	cyan	0.188853933
Xambioona	CC1(C=CC2=C(O1)C=CC(=C2)C3CC(=O)C4=C(O3)C5=C(C=C4)OC(C=C5)(C)C)C	cyan	0.191474608
(2R)-7-hydroxy-2-[4-hydroxy-3-(3-methylbut-2-enyl)phenyl]chroman-4-one	CC(C)=CCc1cc([C@H]2CC(=O)c3ccc(O)cc3O2)ccc1O	cyan	0.213839539
7,4'-Dihydroxyflavone	C1=CC(=CC=C1C2=CC(=O)C3=C(O2)C=C(C=C3)O)O	gray	0.472040636
7-Methoxy-2-methyl isoflavone	CC1=C(C(=O)C2=C(O1)C=C(C=C2)OC)C3=CC=C(C=C3)[N+](=O)[O-]	gray	0.48557006
Nortangeretin	C1=CC(=CC=C1C2=CC(=O)C3=C(C(=C(C(=C3O2)O)O)O)O)O	gray	0.415178823
7-Acetoxy-2-methylisoflavone	CC1=C(C(=O)C2=C(O1)C=C(C=C2)OC(=O)C)C3=CC=CC=C3	gray	0.557448741
7-hydroxy-2-methyl-3-phenyl-chromone	Cc1oc2cc(O)ccc2c(=O)c1-c1ccccc1	gray	0.606643885
Glypallichalcone	COC1=CC=C(C=C1)C(=O)/C=C/C2=C(C=C(C=C2)O)OC	purple	0.47327868
echinatin	COC1=C(C=CC(=C1)O)/C=C/C(=O)C2=CC=C(C=C2)O	purple	0.512427893
Licochalcone B	COC1=C(C=CC(=C1O)O)/C=C/C(=O)C2=CC=C(C=C2)O	purple	0.500556506
licochalcone C	CC(=CCC1=C(C=CC(=C1OC)/C=C/C(=O)C2=CC=C(C=C2)O)O)C	purple	0.378198121
licochalconeD	CC(=CCC1=C(C=CC(=C1)C(=O)/C=C/C2=C(C(=C(C=C2)O)O)OC)O)C	purple	0.343296442
licochalcone E	C[C@H](C1=C(C=C(C(=C1)/C=C/C(=O)C2=CC=C(C=C2)O)OC)O)C(=C)C	purple	0.44542956
licochalcone F	CC(C1=C(C=CC(=C1OC)/C=C/C(=O)C2=CC=C(C=C2)O)O)C(=C)C	purple	0.464481486

licochalcone G	CC(C)(C=C)C1=C(C=C(C(=C1)/C=C/C(=O)C2=C(C=C(C=C2)O)O)OC)O	purple	0.308023033
3,4,3',4'-Tetrahydroxy-2-methoxychalcone	COC1=C(C=CC(=C1O)O)/C=C/C(=O)C2=CC(=C(C=C2)O)O	purple	0.437513047
licochalcone a	CC(C)(C=C)C1=C(C=C(C(=C1)/C=C/C(=O)C2=CC=C(C=C2)O)OC)O	purple	0.356815191
Gancaonin R	CC(=CCC1=C(C(=C(C=C1O)O)CC=C(C)C)CCC2=CC(=C(C=C2)O)O)C	gray	2.080609802
Gancaonin S	CC(=CCC1=C(C(=C(C=C1CCC2=CC(=C(C=C2)O)O)O)CC=C(C)C)O)C	gray	1.631970291
gancaonin T	CC(=CCC1=C(C2=C(C=C1O)OC(C(C2)O)(C)C)CCC3=CC(=C(C=C3)O)O)C	gray	1.206616337
Gancaonin U	CC(=CCC1=C2CCC3=CC(=C(C=C3C2=C(C(=C1O)CC=C(C)C)O)O)O)C	gray	21.90654217
Gancaonin V	CC(=CCC1=C2CCC3=CC(=C(C=C3C2=C(C=C1O)O)O)O)C	gray	21.90654217
2-methyl-5-propyl -nonane	COC1=CC=C(C=C1)C2=COC3=C(C2=O)C=CC(=C3)O[C@H]4[C@@H]([C@H]([C@@H]([C@H](O4)CO)O)O)O[C@H]5[C@@H]([C@](CO5)(CO)O)O	gray	0.974067113
glycyroside	COC1=CC=C(C=C1)C2=COC3=C(C2=O)C=CC(=C3)O[C@H]4[C@@H]([C@H]([C@@H]([C@H](O4)CO)O)O)O[C@H]5[C@@H]([C@](CO5)(CO)O)O	gray	0.974067113
Ononin	COC1=CC=C(C=C1)C2=COC3=C(C2=O)C=CC(=C3)O[C@H]4[C@@H]([C@H]([C@@H]([C@H](O4)CO)O)O)O	gray	0.945931374
Isoononin	COC1=CC2=C(C=C1)C(=O)C(=CO2)C3=CC=C(C=C3)O[C@H]4[C@@H]([C@H]([C@@H]([C@H](O4)CO)O)O)O	gray	0.817136492

Table S2. Related genes analysis of NOS2(pearson correlation coefficient greater than 0.3).

Query	Statistic	P-value	FDR (BH)
NOS2	1	1.00×10^{-20}	1.00×10^{-16}
TIE1	0.45119314	5.25×10^{-20}	5.23×10^{-16}
CDH5	0.441363928	4.05×10^{-19}	2.69×10^{-15}
FLT4	0.431121024	3.18×10^{-18}	1.58×10^{-14}
PLEKHG1	0.429387277	4.47×10^{-18}	1.78×10^{-14}
ERG	0.415765931	6.12×10^{-17}	2.03×10^{-13}
ESAM	0.411947709	1.25×10^{-16}	3.55×10^{-13}
TSPAN18	0.407588082	2.79×10^{-16}	6.94×10^{-13}
NOTCH4	0.406260864	3.55×10^{-16}	7.28×10^{-13}
AOC3	0.405821177	3.84×10^{-16}	7.28×10^{-13}
TMEM204	0.405577683	4.02×10^{-16}	7.28×10^{-13}
RHOJ	0.40430597	5.06×10^{-16}	8.40×10^{-13}
BCL6B	0.403654649	5.69×10^{-16}	8.42×10^{-13}
MMRN2	0.403438591	5.92×10^{-16}	8.42×10^{-13}
CLEC14A	0.403043317	6.35×10^{-16}	8.44×10^{-13}
SOX7	0.399330164	1.23×10^{-15}	1.54×10^{-12}
ZNF521	0.398982097	1.31×10^{-15}	1.54×10^{-12}
EFNB2	0.396880033	1.90×10^{-15}	2.11×10^{-12}
TEK	0.394520963	2.88×10^{-15}	2.96×10^{-12}
PCDH12	0.394351372	2.97×10^{-15}	2.96×10^{-12}
CDH13	0.392546153	4.07×10^{-15}	3.86×10^{-12}
GPR116	0.39205194	4.43×10^{-15}	4.01×10^{-12}
COL15A1	0.388719289	7.87×10^{-15}	6.82×10^{-12}
EMP1	0.388182225	8.63×10^{-15}	7.16×10^{-12}
HECW2	0.386232676	1.20×10^{-14}	9.59×10^{-12}
CALCRL	0.385609345	1.34×10^{-14}	1.02×10^{-11}
SHROOM4	0.382934139	2.10×10^{-14}	1.55×10^{-11}
S1PR1	0.382636995	2.21×10^{-14}	1.57×10^{-11}
LDB2	0.382039909	2.44×10^{-14}	1.68×10^{-11}
CD34	0.381322368	2.75×10^{-14}	1.83×10^{-11}
APOLD1	0.379908584	3.49×10^{-14}	2.24×10^{-11}
ARHGEF15	0.378895908	4.12×10^{-14}	2.57×10^{-11}
MYCT1	0.378591402	4.34×10^{-14}	2.62×10^{-11}
ADAMTSL1	0.374836953	8.04×10^{-14}	4.71×10^{-11}
CYYR1	0.37268289	1.14×10^{-13}	6.50×10^{-11}
PDE2A	0.370286664	1.68×10^{-13}	9.31×10^{-11}
CXorf36	0.367723903	2.54×10^{-13}	1.37×10^{-10}
ROBO4	0.367541952	2.61×10^{-13}	1.37×10^{-10}
NAV1	0.364895773	3.98×10^{-13}	2.03×10^{-10}
DOCK9	0.364613346	4.16×10^{-13}	2.07×10^{-10}
HSPA12B	0.364319746	4.35×10^{-13}	2.12×10^{-10}

SHE	0.36223878	6.04×10^{-13}	2.86×10^{-10}
PLVAP	0.360590155	7.81×10^{-13}	3.62×10^{-10}
ADAMTS7	0.359791115	8.84×10^{-13}	4.00×10^{-10}
ALDH1A3	0.358039853	1.16×10^{-12}	5.13×10^{-10}
COL12A1	0.356295672	1.52×10^{-12}	6.56×10^{-10}
PXDN	0.354817905	1.90×10^{-12}	8.05×10^{-10}
CD93	0.353959971	2.17×10^{-12}	8.84×10^{-10}
USHBP1	0.353935405	2.17×10^{-12}	8.84×10^{-10}
HSPG2	0.352546983	2.68×10^{-12}	1.07×10^{-9}
GAS6	0.352257516	2.80×10^{-12}	1.09×10^{-9}
NOVA2	0.351585277	3.10×10^{-12}	1.19×10^{-9}
KDR	0.351140982	3.32×10^{-12}	1.25×10^{-9}
EDNRB	0.350914387	3.43×10^{-12}	1.25×10^{-9}
ITGB3	0.350855223	3.46×10^{-12}	1.25×10^{-9}
FZD4	0.34978168	4.07×10^{-12}	1.45×10^{-9}
CLEC1A	0.348365841	5.03×10^{-12}	1.73×10^{-9}
FAM70B	0.348343598	5.04×10^{-12}	1.73×10^{-9}
PTPRB	0.347179441	6.00×10^{-12}	1.99×10^{-9}
EXOC3L2	0.346724081	6.42×10^{-12}	2.10×10^{-9}
ANO1	0.34631282	6.82×10^{-12}	2.19×10^{-9}
KIF26A	0.346154652	6.98×10^{-12}	2.20×10^{-9}
REPS2	0.346069953	7.07×10^{-12}	2.20×10^{-9}
EML1	0.345296484	7.92×10^{-12}	2.43×10^{-9}
TMEM233	0.344611442	8.76×10^{-12}	2.65×10^{-9}
GLT8D2	0.344390845	9.05×10^{-12}	2.66×10^{-9}
TAL1	0.344324932	9.14×10^{-12}	2.66×10^{-9}
GJD3	0.344263554	9.22×10^{-12}	2.66×10^{-9}
IGFBP5	0.343873278	9.77×10^{-12}	2.74×10^{-9}
FBN1	0.343871772	9.77×10^{-12}	2.74×10^{-9}
CDC42EP3	0.34335468	1.05×10^{-11}	2.92×10^{-9}
PCDH17	0.343115767	1.09×10^{-11}	2.98×10^{-9}
SLCO2A1	0.342803276	1.14×10^{-11}	3.05×10^{-9}
JAM2	0.342772875	1.15×10^{-11}	3.05×10^{-9}
APLNR	0.342509354	1.19×10^{-11}	3.13×10^{-9}
ARHGAP6	0.341991391	1.29×10^{-11}	3.33×10^{-9}
GNA14	0.341695438	1.34×10^{-11}	3.43×10^{-9}
LRRC70	0.341452975	1.39×10^{-11}	3.51×10^{-9}
C3orf70	0.340718519	1.55×10^{-11}	3.85×10^{-9}
SH2D3C	0.340289067	1.65×10^{-11}	4.05×10^{-9}
SPARC	0.339976924	1.72×10^{-11}	4.19×10^{-9}
C20orf160	0.338586988	2.11×10^{-11}	4.99×10^{-9}
PEAR1	0.338110144	2.26×10^{-11}	5.29×10^{-9}
SLIT3	0.337378563	2.50×10^{-11}	5.80×10^{-9}
ZNF25	0.337233208	2.56×10^{-11}	5.86×10^{-9}

EPHA4	0.337084702	2.61×10^{-11}	5.91×10^{-9}
ARAP3	0.336886835	2.69×10^{-11}	6.02×10^{-9}
FAM124B	0.335659374	3.20×10^{-11}	7.09×10^{-9}
GPR4	0.335567203	3.24×10^{-11}	7.10×10^{-9}
PTPRM	0.335477706	3.29×10^{-11}	7.12×10^{-9}
OLFML2A	0.334667422	3.69×10^{-11}	7.90×10^{-9}
ETS1	0.334533253	3.76×10^{-11}	7.96×10^{-9}
NID2	0.334175533	3.95×10^{-11}	8.29×10^{-9}
SCARF1	0.333960035	4.08×10^{-11}	8.46×10^{-9}
PODXL	0.333471458	4.37×10^{-11}	8.97×10^{-9}
EMCN	0.333341381	4.45×10^{-11}	9.04×10^{-9}
LRRC32	0.332957905	4.70×10^{-11}	9.45×10^{-9}
ELTD1	0.332778773	4.82×10^{-11}	9.59×10^{-9}
ARL15	0.332512847	5.00×10^{-11}	9.86×10^{-9}
ADCY4	0.331865487	5.48×10^{-11}	1.07×10^{-8}
SNRK	0.331344725	5.89×10^{-11}	1.14×10^{-8}
EXOC3L	0.331075082	6.12×10^{-11}	1.17×10^{-8}
SRL	0.329900038	7.21×10^{-11}	1.37×10^{-8}
FHL2	0.32933569	7.80×10^{-11}	1.47×10^{-8}
NOS3	0.328950283	8.23×10^{-11}	1.53×10^{-8}
FAM198B	0.328225699	9.10×10^{-11}	1.68×10^{-8}
SOX17	0.326832224	1.10×10^{-10}	2.02×10^{-8}
ENG	0.325460056	1.33×10^{-10}	2.41×10^{-8}
ZNF423	0.325183838	1.38×10^{-10}	2.48×10^{-8}
FBLN2	0.324888308	1.44×10^{-10}	2.56×10^{-8}
MAP1B	0.324679115	1.48×10^{-10}	2.62×10^{-8}
ZFPM2	0.324533701	1.51×10^{-10}	2.64×10^{-8}
PLAT	0.324330035	1.56×10^{-10}	2.70×10^{-8}
ITGA10	0.324071027	1.61×10^{-10}	2.75×10^{-8}
COL4A1	0.324058541	1.61×10^{-10}	2.75×10^{-8}
UACA	0.323856426	1.66×10^{-10}	2.80×10^{-8}
ARHGEF10	0.323662694	1.70×10^{-10}	2.84×10^{-8}
LOC100126784	0.32359566	1.72×10^{-10}	2.84×10^{-8}
GJA4	0.323583353	1.72×10^{-10}	2.84×10^{-8}
PRR16	0.322382642	2.03×10^{-10}	3.30×10^{-8}
F2R	0.322279872	2.06×10^{-10}	3.30×10^{-8}
SPRY1	0.322257741	2.06×10^{-10}	3.30×10^{-8}
MID2	0.32223224	2.07×10^{-10}	3.30×10^{-8}
THBD	0.321946457	2.15×10^{-10}	3.40×10^{-8}
NOTCH3	0.321619225	2.25×10^{-10}	3.53×10^{-8}
TMOD2	0.31991046	2.83×10^{-10}	4.41×10^{-8}
SYNPO	0.319683714	2.92×10^{-10}	4.51×10^{-8}
TGFB1I1	0.319583642	2.96×10^{-10}	4.54×10^{-8}

PALM2-AKAP2	0.31944978	3.01×10^{-10}	4.58×10^{-8}
ZNF366	0.319401428	3.03×10^{-10}	4.58×10^{-8}
PDGFB	0.31921117	3.11×10^{-10}	4.66×10^{-8}
SASH1	0.318176472	3.57×10^{-10}	5.30×10^{-8}
TCF21	0.318136563	3.59×10^{-10}	5.30×10^{-8}
DPYSL2	0.317662583	3.83×10^{-10}	5.61×10^{-8}
CYTH3	0.317398017	3.97×10^{-10}	5.77×10^{-8}
PDLIM2	0.316595976	4.41×10^{-10}	6.36×10^{-8}
JAM3	0.316556522	4.44×10^{-10}	6.36×10^{-8}
SLC46A3	0.316152003	4.68×10^{-10}	6.66×10^{-8}
TCF4	0.315766974	4.93×10^{-10}	6.96×10^{-8}
LOXL2	0.315712045	4.96×10^{-10}	6.96×10^{-8}
MXRA5	0.315192727	5.31×10^{-10}	7.40×10^{-8}
DLL4	0.314252217	6.02×10^{-10}	8.32×10^{-8}
HEY2	0.313128735	6.97×10^{-10}	9.52×10^{-8}
FAT4	0.31286596	7.22×10^{-10}	9.76×10^{-8}
LAMA2	0.312830928	7.25×10^{-10}	9.76×10^{-8}
LZTS1	0.312772653	7.31×10^{-10}	9.77×10^{-8}
C1orf111	0.31271971	7.36×10^{-10}	9.77×10^{-8}
PSD3	0.312362395	7.71×10^{-10}	1.02×10^{-7}
C1QTNF2	0.312256225	7.82×10^{-10}	1.02×10^{-7}
FSTL1	0.312140886	7.94×10^{-10}	1.03×10^{-7}
RPS6KA2	0.311404117	8.74×10^{-10}	1.12×10^{-7}
KLHL20	0.311174791	9.00×10^{-10}	1.15×10^{-7}
GPR124	0.311039841	9.16×10^{-10}	1.16×10^{-7}
DUSP1	0.310617761	9.68×10^{-10}	1.22×10^{-7}
FGD5	0.310140112	1.03×10^{-9}	1.29×10^{-7}
ZXDA	0.309836113	1.07×10^{-9}	1.33×10^{-7}
COL8A1	0.309446633	1.13×10^{-9}	1.39×10^{-7}
LHX6	0.309249709	1.16×10^{-9}	1.42×10^{-7}
EGFLAM	0.308775144	1.23×10^{-9}	1.50×10^{-7}
MYLK	0.308479844	1.28×10^{-9}	1.55×10^{-7}
FAM26E	0.308376082	1.29×10^{-9}	1.56×10^{-7}
MYOCD	0.307968768	1.36×10^{-9}	1.63×10^{-7}
AKT3	0.307966088	1.36×10^{-9}	1.63×10^{-7}
LOC399959	0.307775916	1.40×10^{-9}	1.66×10^{-7}
KANK3	0.306781736	1.59×10^{-9}	1.85×10^{-7}
EPAS1	0.306594293	1.63×10^{-9}	1.88×10^{-7}
VEGFC	0.306299048	1.69×10^{-9}	1.94×10^{-7}
NPR1	0.306270208	1.69×10^{-9}	1.94×10^{-7}
GPR17	0.30614866	1.72×10^{-9}	1.96×10^{-7}
COL4A2	0.305466103	1.88×10^{-9}	2.12×10^{-7}
RASGRF2	0.30544276	1.88×10^{-9}	2.12×10^{-7}

DCHS1	0.304714326	2.07×10^{-9}	2.31×10^{-7}
ELK3	0.30445636	2.14×10^{-9}	2.38×10^{-7}
COL6A2	0.303264438	2.48×10^{-9}	2.75×10^{-7}
MICAL2	0.303023593	2.56×10^{-9}	2.82×10^{-7}
IGFBP7	0.30273372	2.66×10^{-9}	2.91×10^{-7}
BMX	0.302265543	2.82×10^{-9}	3.05×10^{-7}
CACNA1C	0.302153871	2.86×10^{-9}	3.08×10^{-7}
SNED1	0.302102621	2.87×10^{-9}	3.08×10^{-7}
HEPH	0.301592435	3.07×10^{-9}	3.27×10^{-7}
FXYD6	0.301316226	3.17×10^{-9}	3.35×10^{-7}
PLCL1	0.301302068	3.18×10^{-9}	3.35×10^{-7}
CALD1	0.300709703	3.42×10^{-9}	3.58×10^{-7}
TRO	0.300685925	3.43×10^{-9}	3.58×10^{-7}
MYH11	0.300448226	3.54×10^{-9}	3.67×10^{-7}
SLC41A2	0.300019199	3.73×10^{-9}	3.83×10^{-7}
